# The role and mechanism of p53 F229V mutation in inhibiting pseudorabies virus replication

**DOI:** 10.3389/fmicb.2025.1628916

**Published:** 2025-11-26

**Authors:** Cuilian Yu, Jingshuai Zhang, Shumin Chen, Zhao Wang, Liqi Zhu, Kezhou Wang

**Affiliations:** 1 School of Laboratory Animal & Shandong Laboratory Animal Center, Shandong First Medical University & Shandong Academy of Medical Sciences, Jinan, China; 2 Veterinary College, Yangzhou University, Yangzhou, Jiangsu, China; 3 Shandong Provincial Center for Animal Disease Control and Prevention (Zoonoses Surveillance Center of Shandong Province), Jinan, China

**Keywords:** p53, pseudorabies virus, p53 mutation, F229V, virus

## Abstract

The p53 protein is a key transcription factor that regulates cellular responses to stress and is widely recognized as a host restriction factor against various viral infections. However, its specific role in pseudorabies virus (PRV) replication, pathogenesis, and host response remains unclear. This study identified a swine p53 (sp53) mutation in the PK15 cell line under prolonged passage stress, characterized by an amino acid substitution at position 229, replacing phenylalanine with valine (sp53 F229V). This mutation eliminates the transcriptional activity of wild-type p53 and confers resistance to PRV infection. Notably, it reverses p53’s original pro-viral role, converting it into an inhibitor of PRV proliferation. Further analysis revealed that the PRV early protein EP0 promotes the degradation of sp53 F229V through the proteasome pathway. These findings indicate that a defined p53 alteration can decouple transcriptional and antiviral functions in a mutation-specific, context-dependent manner. The EP0–p53 interface emerges as a candidate target to modulate PRV replication, pending validation. These findings were obtained *in vitro* and require *in vivo* validation in pigs to determine their relevance to PRV pathogenesis.

## Background

Pseudorabies virus (PRV), a member of the *Varicellovirus* genus within the *Herpesviridae* subfamily, is the causative agent of pseudorabies in pigs (Mettenleiter, 2000; Pomeranz et al., 2005). The PRV genome is a large, linear, double-stranded DNA molecule approximately 150 kb in length, with a GC content of 73%, encoding 70–100 distinct viral proteins (Ren et al., 2023; Zhang et al., 2024). PRV is primarily transmitted through oral-nasal contact, airborne droplets, and semen. It predominantly replicates in respiratory epithelial cells, monocytes, and the central nervous system of pigs, leading to severe health issues (Chen and Yu, 2024; Bude et al., 2024). In neonatal piglets, PRV infection often causes encephalitis, pneumonia, and other respiratory and nervous system disorders, while in sows, it is associated with reproductive problems (Tang et al., 2022; Sun et al., 2023). Recently, emerging PRV variants have evaded protection provided by traditional vaccines, resulting in highly pathogenic strains that pose significant threats in certain regions (Pan et al., 2024). Despite advances in understanding PRV pathogenesis, the interaction mechanisms between PRV and host antiviral immune proteins remain poorly understood. Further research in this area is essential for developing effective prevention and control strategies for PRV.

The p53 gene, often called the “guardian of the genome,” is crucial for maintaining genomic stability in multicellular organisms (Hao et al., 2019; Janic et al., 2025). It orchestrates cellular stress responses by regulating the expression of hundreds of genes, including those involved in apoptosis, DNA repair, and immune responses (Oren and Rotter, 2010; Yang et al., 2025; Tong et al., 2024). These functions are critical for cancer prevention. Mutations in p53 are observed in half of human cancers, and most of these mutations result in the loss of tumor-suppressive functions (Chen et al., 2022). Current studies on mutant p53 primarily focus on its gain-of-function roles, which include promoting cancer progression, drug resistance, and immune evasion (Efe et al., 2024). Approximately 75% of p53 mutations result in the loss of tumor-suppressive functions, allowing mutant p53 to actively contribute to cancer progression through mechanisms such as mTOR suppression, Cip1 inhibition, and NF-κB pathway activation (Marei et al., 2021; Aleksandrova et al., 2024).

Viral infections, as a form of cellular stress, can activate p53-mediated responses, leading to apoptosis in infected cells and suppression of viral replication (Muñoz-Fontela et al., 2008; Jana et al., 2024). In agreement with these observations, p53 status correlates with replication outcomes *in vitro* across diverse viruses: knockout/knockdown often increases replication, whereas overexpression tends to decrease replication in cell culture for hepatitis C virus, vesicular stomatitis virus, Sendai virus, poliovirus, influenza A virus, vaccinia virus, human papillomavirus, and JC virus (Muñoz-Fontela et al., 2008). Many viruses therefore encode factors that neutralize p53-dependent defenses, including ubiquitin–proteasome–linked turnover mediated by ICP0/EP0-like activities (Isaacson and Ploegh, 2009). At the same time, exceptions have been reported: p53 loss can impair replication or gene expression for human cytomegalovirus and HSV-1 in some systems (Maruzuru et al., 2016), and p53 is required for efficient expression of the Epstein–Barr virus immediate-early regulator BZLF1 (Kraus et al., 2020). Together, these observations indicate that p53 acts in context- and stage-dependent manner during infection—early stabilization/co-option versus later antagonism—providing the rationale to examine how a defined p53 variant shapes alphaherpesvirus replication *in vitro* (Xu et al., 2022).

In PK15 cells subjected to long-term passaging stress, we identified a novel p53 mutant, F229V, characterized by a phenylalanine-to-valine substitution at position 229. This mutation eliminates the transcriptional activity of wild-type p53 while conferring antiviral capabilities, particularly against PRV. Our findings indicate that sp53 F229V inhibits PRV replication and enhances the environmental adaptability of the mutant cell lines. Further investigation revealed that the PRV early protein EP0 promotes the degradation of sp53 F229V through the proteasome pathway, thereby neutralizing its antiviral effects. These results deepen our understanding of p53 mutations in host-virus interactions and suggest potential therapeutic strategies for viral diseases and cancers. Future studies should aim to uncover the precise molecular mechanisms underlying these interactions to advance antiviral and anticancer therapies. Building on this rationale, we asked whether a defined p53 variant could decouple canonical transcriptional activity from effects on virus replication. We therefore focused on sp53 F229V—identified in long-term passaged porcine cells—to examine how specific p53 alterations shape alphaherpesvirus replication *in vitro*.

## Materials and methods

### Cell culture and viruses

PK15 cells (porcine kidney cells, ATCC CCL-33), Vero cells (ATCC CCL-81), 3D4/21 cells (porcine alveolar macrophages, ATCC CRL-2843) and HCT116 (ATCC CCL-247) p53^−/−^ cells were cultured in Dulbecco modified Eagle medium (DMEM). Primary porcine hepatocytes were isolated from liver lobes of 6-8-week-old SPF Large-White piglets by collagenase-P perfusion as described by Meng et al. (2010). Porcine Embryonic Fibroblasts (PEFs) cells were derived from 25-35-day Large-White embryos via trypsin digestion (Park et al., 2022). Primary cells were used between passages 1 and 15 for all experiments. All cells were cultured in medium supplemented with 10% (vol/vol) fetal bovine serum (FBS), 1% penicillin–streptomycin, and 1% glutamine and maintained in a humidified incubator with 5% CO_2_ at 37 °C.

PRV BarthaK61 strain vaccine was purchased from Weike Biotech Co, Harbin, China. The virus has been plaque-purified and adapted to PK15 cells, subsequently propagated exclusively in PK15 cells (passage 3–5 after purification) to maintain genetic stability. The master virus stock was aliquoted and stored at −80 °C; working stock was generated from a single aliquot with ≤ 2 additional passages to avoid attenuation.

Virus titer was determined in duplicate by plaque assay on Vero cells as described in Methods; final working stock concentration: 2.4 × 10^7^ PFU mL^−1^ (mean of three independent assays). All infections were performed at the indicated multiplicity of infection (MOI) calculated from this titer. Passage history and thaw cycles were recorded to ensure ≤ 5 cumulative passages from the original vial for any experiment.

All sgRNAs were designed using the online CRISPR Design Tool,^1^ and target the amino-terminal regions of the PRV Early Protein 0 (EP0) open reading frames. The CRISPR/Cas9 targeting plasmids were assembled by cloning annealed oligonucleotides encoding the desired sgRNAs into the BbsI sites of pUC19-sgRNA vector (Addgene #51132) that carries the guide-RNA scaffold sequence; Cas9 was expressed from pcDNA3.1-Cas9 (Addgene #41815, sequence-verified). The RFP cassette (714 bp) was amplified from pmRFP-C1 (Clontech) with primers RFP-fwd 5′-AATAGTAATCAATTACGGGGTCATT-3′ and RFP-rev 5′-AGATACATTGATGAGTTTGGACAAACCA-3′ (annealing 58 °C, 30 s) using Q5 HF polymerase (NEB). The EP0 bait sequence in the donor plasmid, which is the short fragment of EP0 locus encompassing the targeting sequences of sgRNAs, was amplified from the viral DNA using the following primers: EP0-fwd: 5′-GACTGCCCCATCTGTCTG-3′, EP0-rev: 5′-CCGTAATTGATTACTATTTCCTCGGTATAGTCTTCACCC-3′. The fusion fragment of RFP expression cassette with the EP0 bait sequence at its 5′-end was generated using High-Fidelity DNA polymerase, and then inserted into the pCloneEZ- Blunt-Amp/HC cloning vector. All constructs were verified by sequencing. For generation of mutant viruses, mock or sgRNA constructs (0.5 μg), pCDNA3.1 cas9 (0.5 μg) expressing Cas9 and viral DNA genome (1 μg) were co-transfected into PK15 cells using Lipofectamine 2000 (Invitrogen, USA) according to the manufacturer’s instructions. 2–4 days after transfection, the cells with expected cytopathic effect (CPE) were collected for genotyping analysis by PCR. The supernatants of cells with CPE were serially diluted by 10^−1^–10^−8^ fold, and inoculated into newly plated PK15 cells. 3–4 days later, viral genomic DNA was extracted and purified from PK15 cells following a standard protocol. PCR was performed using sequence-specific primer pairs (for EP0: forward,5′-CGCAGCGCCGCTTTCAGACCCA-3′ and reverse,5′ -GGAGCATGGCCTCGGTCAC-3′). After the purification of amplified DNA, the EP0 fragment were cloned into PMD18-T vector and transformed into *E. coli* DH5α (Thermo, 18265017). The colonies containing the inserted genes were sequenced. Mutants were identified by comparison to the wild-type sequence (Xu et al., 2015; Qin et al., 2019; Yu et al., 2020).

### Antibodies and reagents

The following primary antibodies were used: mouse monoclonal anti-p53 (DO-1, sc-126, Santa Cruz Biotechnology, Santa Cruz, CA, USA), rabbit polyclonal anti-HA (Y-11, sc-805, Santa Cruz Biotechnology), mouse monoclonal anti-Tubulin (DM1A, M1765, MBL, Beijing, China), and mouse monoclonal anti-Flag (M2, F1804, Sigma, St. Louis, MO, USA). The antibodies against PRV TK, PRV US3, PRV EP0, PRV gD, PRV VP5 and PRV UL42 were described previously (Qin et al., 2019; Yu et al., 2020). MG132 was purchased from APExBIO. Flag M2 beads (A2220) were purchased from Sigma. Sodium dodecyl sulfate (SDS), DL-Dithiothreitol (DTT), puromycin and bovine serum albumin (BSA) fractions were purchased from Amresco Biotechnology.

### Plasmids and transfection

Sp53-WT and sp53-F229V cDNAs were amplified by PCR using cDNAs made from PEF cells and PK15 cells as templates, respectively. Then cloned into Flag-/pRK5 vector. The PRV EP0 and UL12 genes were amplified from the PRV Bartha K61 genome and then cloned into Flag-/pRK5 vector. Plasmids encoding HA-ubiquitin were described previously (Lang et al., 2020; Tang et al., 2006). For constructions of luciferase reporters of various p53 target genes, forward and the complementary reverse oligonucleotides which include the corresponding p53 response element were annealed in 1x annealing buffer (95 °C 5 min, 0.1 °C/s, to 25 °C) and the resulting double-stranded fragment was cloned into pBV-Luc (addgene Plasmid #16539). All the constructs were confirmed by DNA sequencing. The sequence of primers used for luciferase assay and plasmids construction were provided in Supplementary Table S1.

Plasmids were transfected into PK15 cells using Lipofectamine LTX reagent (Invitrogen) according to the corresponding manufacturer’s protocol. PK15 cells (1 × 10^5^/well, 12-well plate) were transfected with 500 ng plasmid + 1.5 μL Lipofectamine LTX + 1 μL PLUS in 200 μL Opti-MEM for 5 h, then medium was replaced; empty Flag-pRK5 served as mock control. Expression was verified by Western blot (anti-Flag, 24 h post-transfection). All inserts were Sanger-sequenced.

### Virus infection and plaque assay

Cells were infected with PRV WT or PRV-EP0 KO viruses with the indicated multiplicity of infection (MOI = 0.01, 0.1, 1, 5) for 1 h, followed by washes with PBS and incubation in complete DMEM supplemented with 5% FBS for the indicated durations showed in Figure legend. For viral propagation, PK15 cells were infected with PRV WT or PRV-EP0 KO viruses at an MOI of 0.1. After 1 h, the cells were washed with PBS, supplemented with DMEM containing 5% serum, and further cultured for 72 h. Subsequently, the cells underwent three freeze–thaw cycles before centrifugation to collect the supernatant. For the MG132 (ApexBio) treatment, a final concentration 20 μM of MG132 was added into culture medium at 1 h post infection to allow efficient viral entry.

Viral yields were determined by plaque assay on Vero cells performed in triplicate technical wells and repeated in three independent biological replicates. Vero cells were seeded in 6-well plates at a density of 2 × 10⁵ cells per well and incubated until ~90% confluence. Serial 10-fold dilutions of the virus stock were prepared in DMEM without FBS. 500 μL of each dilution was added to the wells and incubated at 37 °C for 1 h with gentle agitation every 10 min. After adsorption, the inoculum was removed, and cells were overlaid with 3 mL of a 1:1 mixture of 2 × DMEM and 2% low-melting agarose, yielding a final concentration of 1 × DMEM containing 1% agarose. The plates were incubated for 3–4 days, and plaques were visualized by staining with 0.5% neutral red for 4–6 h.

### Generation of PK15 (p53^−/−^) cells

PK15 cells were seeded into each well of a 6-well plate to achieve 70% confluency and were transfected with CAS9 and sgRNA containing a target sequence complementary to the exonsa 1–3 of p53, and 48 h later, the cells were subjected to a limiting-dilution cloning by serial dilution and seeded into 96-well plate at 0.5 cells/well in complete DMEM. Colonies derived from single-cell wells were expanded until enough cells were available for total protein extraction and detecting p53 expression by Western blot. Genomic DNA was extracted and identified from p53-negative clones as determined by Western blot. A 478 or 610-bp region flanking the target was PCR-amplified (Q5, 98 °C 30 s; 35 × [98 °C 10 s, 60 °C 20 s, 72 °C 30 s]; 72 °C 2 min). Cas9 had been delivered as pLV3-U6 plasmid (Addgene #51133) pX459 plasmid (Addgene #62988). Amplicons were Sanger-sequenced on an ABI 3730xl (Sangon Biotech) to verify indels.

The PCR primers used in this experiment are shown in Supplementary Table S2.

### Western blot

Whole-cell lysates were prepared in lysis buffer (50 mM Tris-Cl [pH 8.0], 150 mM NaCl, 1.0% Triton X-100, 10% glycerol, 20 mM NaF, 1 mM DTT, and 1 × complete protease mixture). Protein concentration was determined using the Bradford assay. Equal amounts of protein (20 μg per lane) were separated by 10% SDS-PAGE and transferred to a nitrocellulose membrane at 100 V for 1 h. Membranes were blocked with 5% non-fat milk in PBST for 1 h at room temperature, followed by incubation with primary antibodies (anti-p53 1:1000, anti-US3 1:1000, anti-TK 1:1000, anti-gD 1:1000, anti-EP0 1:500, anti-VP5 1:1000, anti-UL42 1:1000, anti-Flag 1:2000, anti-HA 1:2000, anti-Tubulin 1:5000 loading control) in 5% BSA-PBST at 4 °C overnight, then HRP-conjugated secondary antibody (goat anti-mouse, 1:10000) for 45 min at room temperature. Bands were visualized with ECL (30–60 s exposure) on a Tanon-5200 workstation.

### Real-time PCR

Total RNAs were extracted using TRIzol (Invitrogen) according to the manufacturer’s protocol. A total of 0.8 μg of RNA from different treatments was reversely transcribed into cDNA using M-MLV reverse transcriptase (Promega) with an oligo(dT)18 primer. Real-time PCR was performed using an UltraSYBR Mixture (Beijing CoWin Biotech, Beijing, China) on a ViiA 7 real-time PCR system (Applied Biosystems). Sample data were normalized to Glyceraldehyde-3-phosphate dehydrogenase (GAPDH) expression using the 2^(−ΔΔCt) method. The following gene-specific primers in Supplementary Table S3 were used for RT-PCR assays.

### siRNA transfection

Transfection procedure was performed according to Lipofectamine RNAiMAX (Invitrogen) for the instructions. Sp53 siRNA: 5’-CUACUUCCUGAAAACAACG-3′ was synthesized by Sangon Biotech; PK15 cells were transfected with 50 nM siRNA for 48 h, and knock-down efficiency was verified by Western blot (anti-p53) and/or qPCR.

### Ubiquitination experiment

PK15 cells were transfected with Flag-sp53 and HA-Ub plasmids. After 24 h, cells were infected with PRV WT or EP0 KO strains (MOI = 1). Twelve hours post-infection, cells were treated with 20 μM MG132 for 6 h, lysed in RIPA buffer (50 mM Tris–HCl pH 7.5, 150 mM NaCl, 1% NP-40, 0.5% DOC, 0.1% SDS, 1 × protease inhibitor, 20 mM NEM). Lysate containing 1 mg of protein was pre-cleared with mouse IgG beads, then incubated with anti-Flag M2 magnetic beads. Beads were washed 4×, eluted in 1 × SDS sample buffer, and analyzed by Western blot: input (5%), IP fractions probed with anti-HA (1:2000) for ubiquitin smears and anti-Flag-HRP (1:5000) for loading. Ubiquitin signal was quantified with ImageJ and normalized to Flag immunoprecipitated protein.

### Luciferase reporter gene analysis

Luciferase reporter assay was carried out in 24-well plates (5 × 10^4^ HCT116 p53^−/−^ cells/well). Each well received 310 ng total DNA: 200 ng firefly luciferase reporter, 10 ng pRL-TK (Renilla), and either 100 ng Flag-p53 (WT or F229V) or empty Flag-pRK5 as mock control, complexed with 0.8 μL Lipofectamine 2000 in 50 μL Opti-MEM. Firefly and Renilla activities were measured 24 h post-transfection using the Dual-Luciferase Reporter Assay System (Promega) on a Tecan Spark reader; firefly values were normalized to Renilla (F/R ratio) and expressed relative to mock (set to 1). Data are mean ± SD of three independent experiments (*n* = 3 wells each). Statistical significance was assessed by one-way ANOVA followed by Tukey’s post-hoc test (GraphPad Prism 9); *p* < 0.05 was considered significant.

### Statistical analysis

Data are presented as mean ± SD from at least three independent experiments. Statistical significance was assessed with two-tailed unpaired Student’s t-test or one-way ANOVA followed by Tukey’s post-hoc test using GraphPad Prism 9; *p* < 0.05 was considered significant (*p < 0.05, **p < 0.01, ***p < 0.001).

## Results

### The sp53 F229V loses transcriptional activity

**Figure 1:**
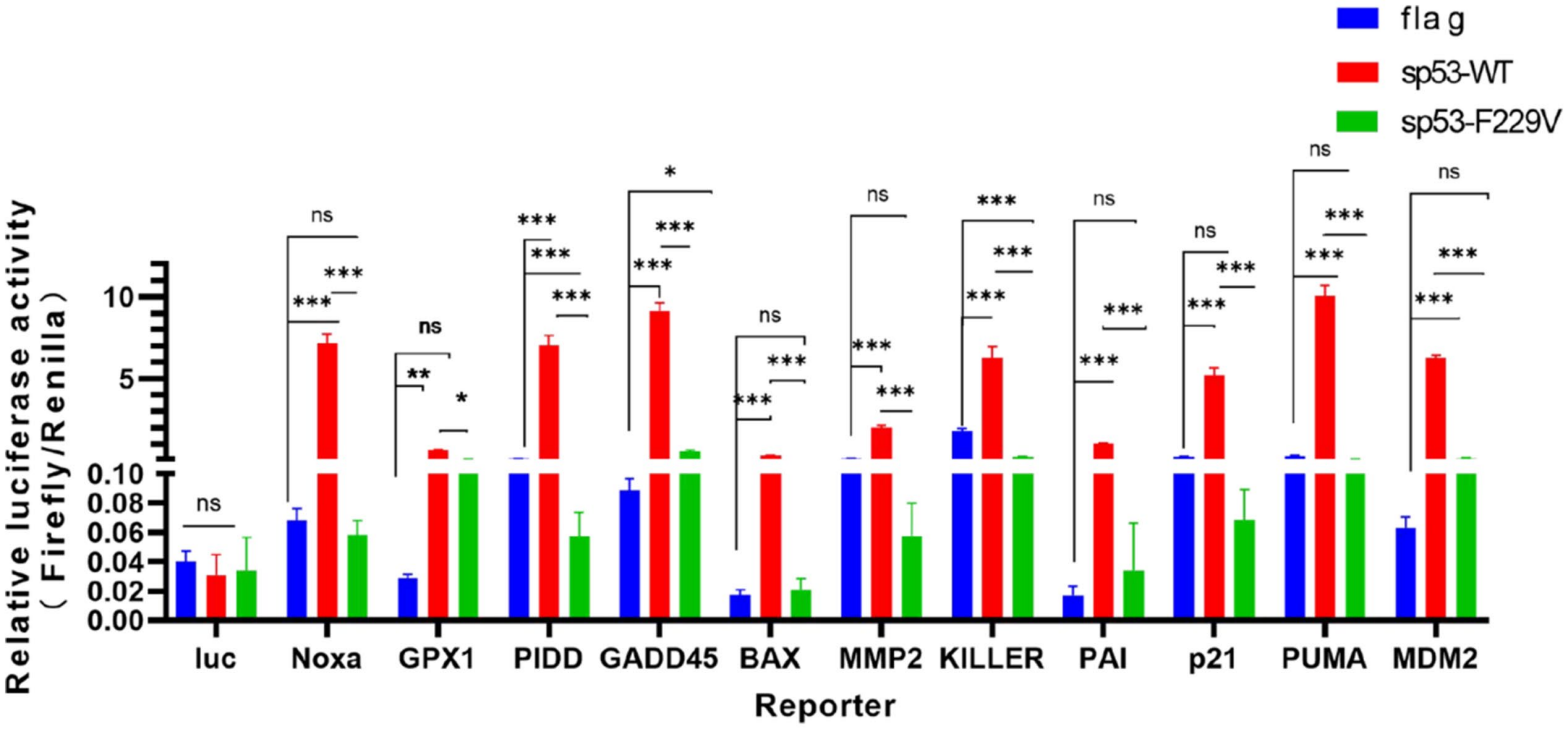
The sp53 F229V loses transcriptional activity. P53 target gene luciferase reporters were co-transfected with empty vector or Flag-p53 (WT or F229V) into HCT116 (p53−/−) cells, with renilla reporter for normalization. Reporter activity was measured. Data are presented as mean ± SD (*n* = 3 independent experiments). Statistical significance was determined using a two-tailed t-test (*p < 0.05; **p < 0.01; ***p < 0.001).

In long-term passaged PK15 cells, a naturally occurring mutation in the sp53 gene was identified, where phenylalanine at position 229 was substituted with valine, resulting in the F229V variant. The transcriptional activity of wild-type sp53 (sp53 WT) and mutant sp53 (sp53 F229V) was evaluated using luciferase reporter assays targeting classical p53-regulated genes. The results showed that sp53 F229V had a significant loss of transcriptional activity across multiple gene categories. For instance, cell cycle-related genes, p21/CDKN1A (Cyclin-Dependent Kinase Inhibitor 1A); GADD45 (growth arrest and DNA-damage-inducible 45) and apoptosis-related genes, PUMA/BBC3 (BCL2 binding component 3); NOXA/PMAIP1 (phorbol-12-myristate-13-acetate-induced protein 1) exhibited over a 90% reduction in activation compared to sp53 WT. Similarly, antioxidant-related genes, GPX (Glutathione Peroxidase); SERPINE1/PAI-1 (Plasminogen Activator Inhibitor 1) demonstrated substantial decreases in transcriptional activation (mean ± SD, *n* = 3, *p < 0.05, **p < 0.01, ***p < 0.001, Figure 1). These results highlight the severe impairment of transcriptional regulation caused by the sp53 F229V mutation.

### The sp53 F229V mutation inhibits PRV replication

**Figure 2:**
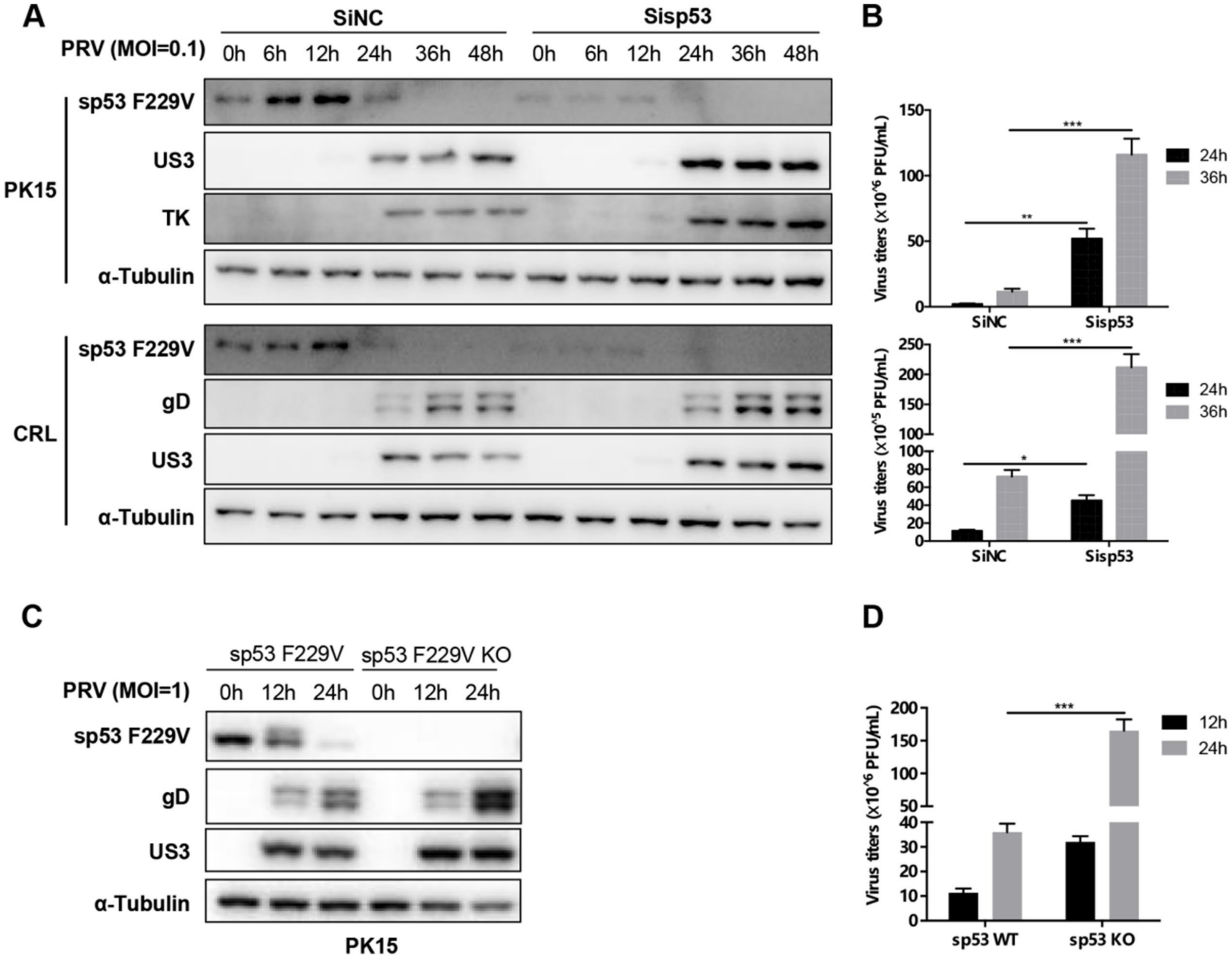
The sp53 F229V mutation inhibits PRV replication. **(A)** PK15 and CRL cells were transfected with either a non-targeting control siRNA (Si-NC) or an siRNA targeting sp53 (Si-sp53) for 24 h, followed by infection with PRV WT (MOI = 0.1). Cells were collected at 6-, 12-, 24-, 36-, and 48- h post-infection for Western blot analysis of sp53 and viral protein expression. **(B)** Viruses released in supernatants at 24- and 36- h post-infection were assessed by plaque assay. **(C)** PK15 cells with sp53 F229V expressing or not (sp53 F229V KO) were infected with PRV WT (MOI = 1). Cells were collected at 12- and 24- h post-infection for Western blot analysis of sp53 and viral proteins. **(D)** Supernatants were collected at the same time points for plaque assays. The data represent the means ± the SD of three independent experiments. Statistical analyses were performed by ANOVA, using GraphPad Prism software (*, *p* < 0.05; **p < 0.01; ***, *p* < 0.001).

To investigate the impact of the sp53 F229V mutation on PRV replication, we compared PRV replication in two porcine cell lines that endogenously carry sp53 F229V (PK15 and CRL). In PK15, sp53 was modulated either by siRNA-mediated knockdown or by CRISPR–Cas9-mediated knockout, performed in separate experiments; in CRL, sp53 was modulated by siRNA knockdown only. Viral protein accumulation and PRV titers were used as readouts *in vitro*. PRV infection (MOI = 0.1) in sp53-knockdown cells led to a ~ 2-fold elevation in viral protein expression (Western blot, Figure 2A; Supplementary Figure S3) and an approximate one-log elevation in viral titer (plaque assay, Figure 2B), indicating enhanced replication. Similarly, deletion of sp53 F229V significantly augmented PRV replication capacity (Figure 2C), leading to ~4.5 times more viral plaques than cells endogenously expressing sp53 F229V (Figure 2D). These results demonstrate that the sp53 F229V limits PRV replication *in vitro*, maintaining a degree of antiviral activity absent in sp53 F229V-KO cells.

### Wild-type sp53 promotes PRV replication

**Figure 3:**
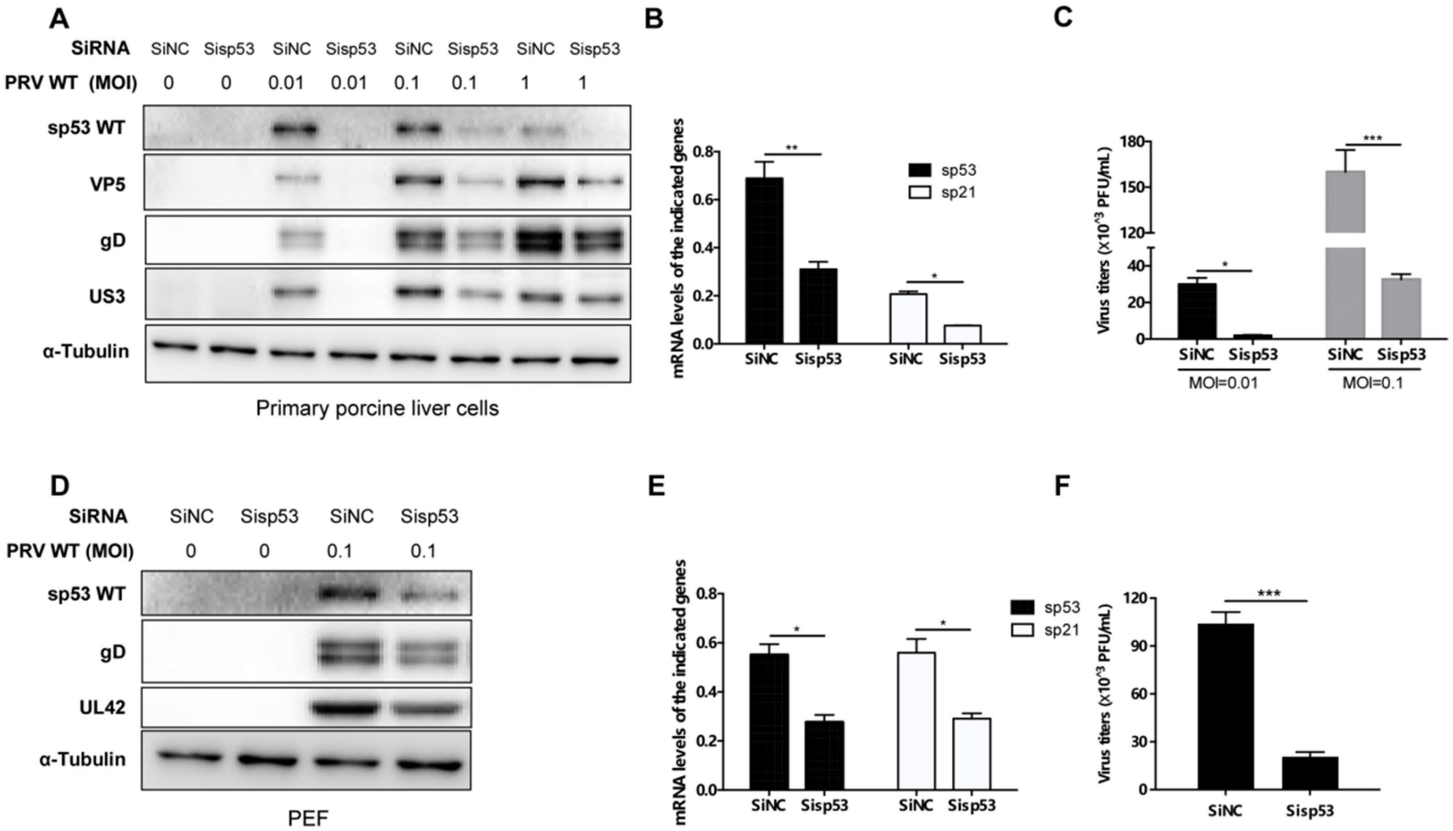
Wild-type sp53 promotes PRV replication. **(A–C)** Primary porcine liver cells transfected with SiNC or Si-sp53 for 24 h, a subset of cells was harvested for RNA extraction, and the mRNA levels of sp53 and sp21 were quantified by qRT-PCR **(B)**. The remaining cells were infected with PRV WT (MOI = 0.01, 0.1 or 1) for 24 h, Western blot was performed to analyze the expression of sp53 and viral proteins **(A)**, while supernatants from cells infected with PRV WT (MOI = 0.01 and 0.1) were collected for plaque assays **(C)**. **(D–F)** Primary porcine embryonic fibroblasts cells (PEF) transfected with SiNC or Si-sp53 for 24 h, a subset of cells was harvested for RNA extraction, and the mRNA levels of sp53 and sp21 were analyzed by qRT-PCR **(E)**. The remaining cells were infected with the PRV WT (MOI = 0.1) for 24 h, Western blot was used to detect the expression of sp53 and viral proteins **(D)**, while supernatants were collected for plaque assays **(F)**. The data represent the means ± the SD of three independent experiments. Statistical analyses were performed by ANOVA, using GraphPad Prism software (*p < 0.05; **p < 0.01; ***p < 0.001).

To study the impact of wild-type sp53 on PRV infection, primary porcine liver cells and embryonic fibroblasts were used as experimental models. The transcriptional activity of wild-type sp53 was confirmed by analyzing the expression of its downstream target gene, p21. Knocking down wild-type sp53 significantly reduced p21 transcription levels (Figures 3B,E), validating its functional activity in these cells. Following PRV infection, sp53 knockdown leads to about a twofold decrease in viral protein expression (Figures 3A,D; Supplementary Figure S4). The viral titer declines over 5 - fold in cells with p53 knockdown (Figures 3C,F). These results indicate that wild-type sp53 expression was associated with higher viral protein accumulation and PRV titers in vitro.

### PRV infection degrades mutant sp53

**Figure 4:**
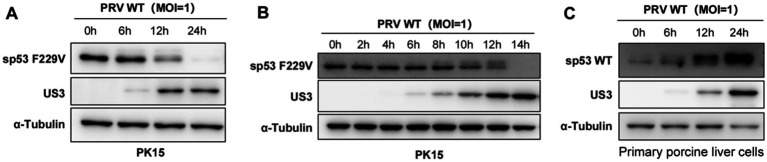
PRV infection degrades mutant sp53. **(A)** PK15 cells were infected with PRV WT (MOI = 1), and cells were harvested at 6-, 12- and 24- h post-infection for Western blot analysis of sp53 and viral proteins. **(B)** PK15 cells were infected with PRV WT (MOI = 1), and cells were harvested at 2-, 6-, 8-, 10-, 12-, and 14- h post-infection for Western Blot analysis of sp53 and viral protein expression. **(C)** Primary porcine liver cells were infected with PRV WT (MOI = 1), and cells were harvested at 6-, 12-, and 24- h post-infection for Western Blot analysis of sp53 and viral proteins expression.

To investigate the effect of PRV infection on the wild-type and mutant sp53, we analyzed the protein levels of sp53 in PRV-infected PK15 (Figures 4A,B) and primary porcine liver cells (Figure 4C) over time. The results of Western blotting showed that within 24 h post - PRV infection (MOI = 1), the protein level of wild - type sp53 gradually increased (Figure 4C). In contrast, PRV infection (MOI = 1) led to a gradual decrease in p53 F229V protein levels, becoming almost undetectable by 14 h post-infection (Figures 4A,B; Supplementary Figure S5). These findings suggest that PRV utilizes different strategies to modulate sp53 proteins: stabilizing wild-type sp53 to support viral replication, while degrading the sp53 F229V mutant, which exerts antiviral effects.

### PRV early protein EP0 is involved in the degradation of mutant sp53

The PRV early protein EP0 shares homology with the HSV-1 ICP0 protein, particularly in its conserved RING domain. Based on this similarity, we hypothesized that EP0 contributes to the ubiquitination and degradation of sp53 F229V and neutralizes the inhibitory effects of sp53 F229V on viral replication. To test this hypothesis, we compared the effects of PRV WT and PRV EP0 KO strains on sp53 F229V protein levels and assessed the impact of EP0 overexpression on endogenous sp53 F229V degradation.

**Figure 5:**
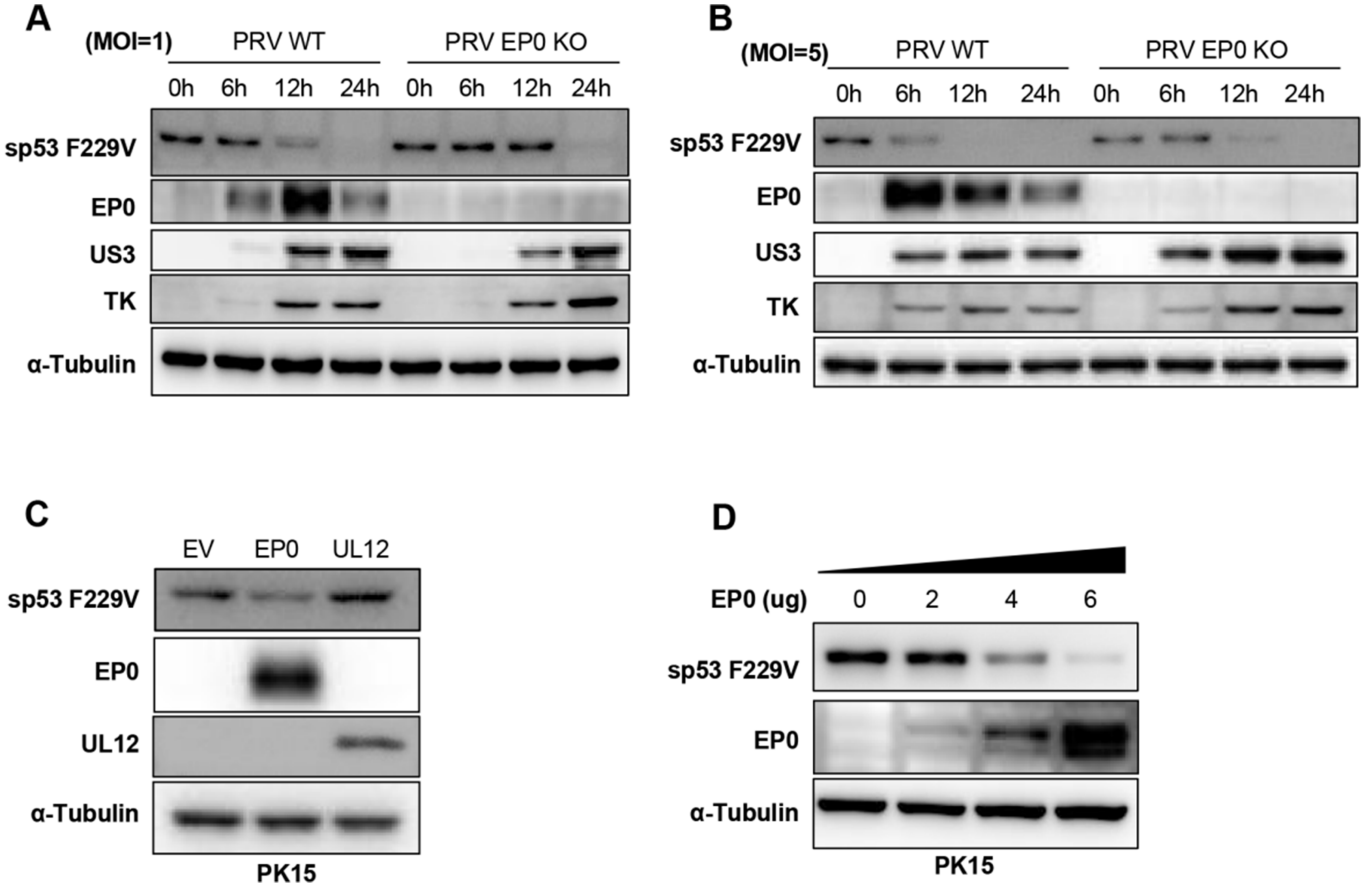
PRV early protein EP0 is involved in the degradation of mutant sp53. **(A,B)** PK15 cells were infected with PRV WT or PRV EP0 KO at MOI = 1 **(A)** or MOI = 5 **(B)**, cells were harvested at 6-, 12- and 24- h post-infection, and Western blot was performed to analyze the expression levels of sp53 and viral proteins. **(C,D)** PK15 cells were transfected with Flag-EP0 or Flag-UL12 expressing plasmids **(C)** or increasing concentrations of Flag-EP0 expression plasmid **(D)**. After 24 h, Western blot was used to detect the expression levels of endogenous sp53, Flag-EP0, Flag-UL12, and α-Tubulin.

The results showed that PRV WT infection led to significantly faster degradation of sp53 F229V compared to PRV EP0 KO strains, as demonstrated at different MOI levels (Figures 5A,B). Furthermore, overexpression of EP0 markedly reduced endogenous sp53 F229V levels, while overexpression of another viral protein, UL12, had minimal effect (Figure 5C). Additional experiments revealed that EP0-mediated downregulation of sp53 was dose-dependent (Figure 5D).

These findings demonstrate that EP0 involved in the degradation of sp53 F229V, thereby neutralizing its antiviral effects. This mechanism likely enables PRV to evade p53-mediated host defenses and promote viral replication.

### EP0 degrades mutant sp53 through the proteasome pathway

**Figure 6:**
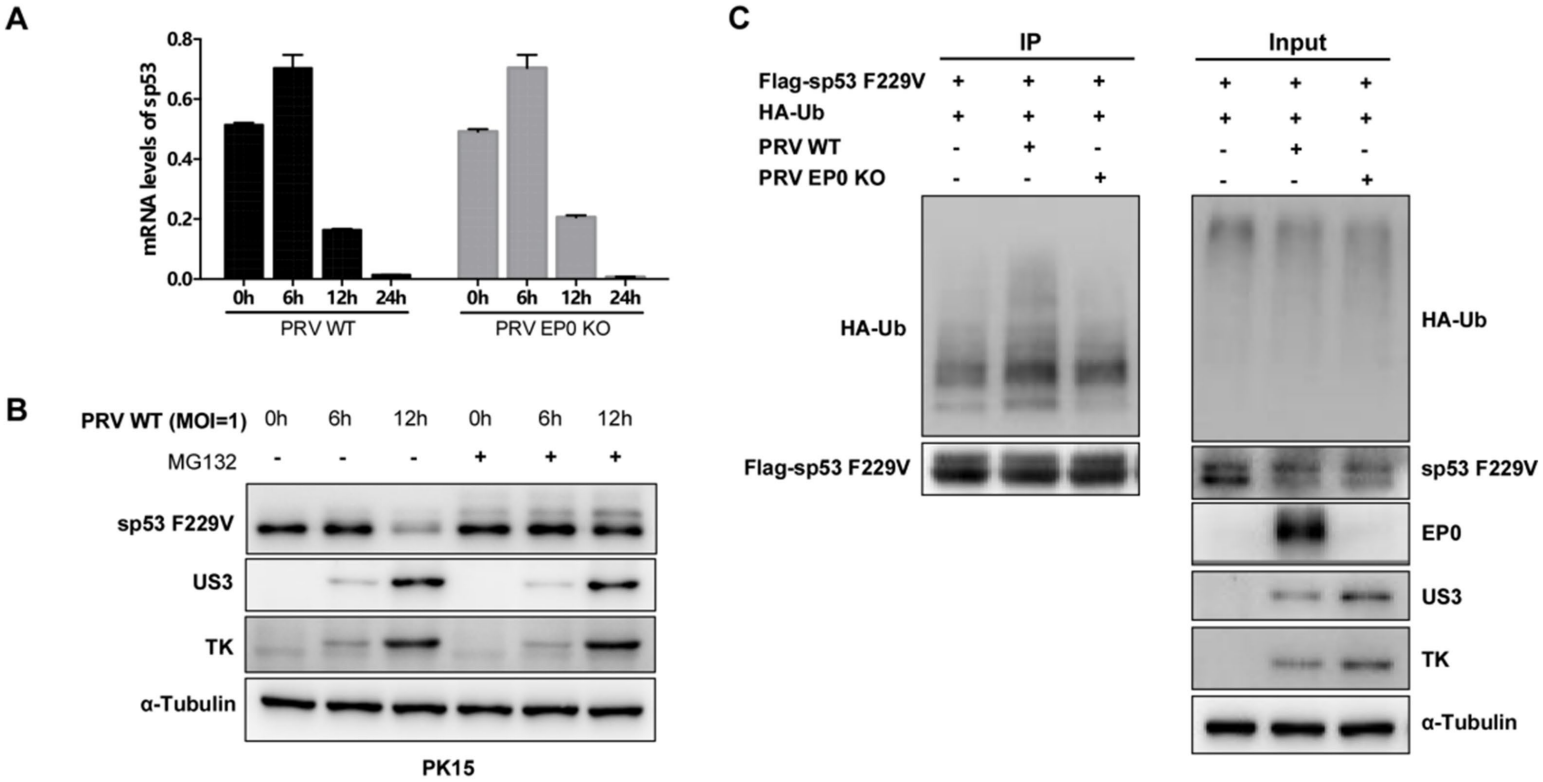
EP0 degrades mutant sp53 through the proteasome pathway. **(A)** PK15 cells were infected with PRV WT (MOI = 1) or PRV EP0 KO (MOI = 1), and RNA was extracted at 6-, 12- and 24- h post-infection for qRT-PCR analysis of sp53 mRNA levels. GAPDH was used as the internal control. **(B)** Western blot was performed to analyze the expression levels of sp53 and viral proteins in PK15 cells infected with PRV WT (MOI = 1) for the indicated time. Cells were treated with DMSO or MG132 for 5 h before harvested for Western blot analysis. **(C)** PK15 cells were transfected with Flag-sp53 and HA-Ub expression plasmids for 24 h and then infected with PRV WT or PRV EP0 KO (MOI = 1). At 12 h post-infection, cells were collected for immunoprecipitation (IP) using M2 beads, followed by Western blot analysis to assess the ubiquitination levels of sp53. All experiments were performed in triplicate. Data are presented as mean ± SD, and statistical significance was determined using GraphPad Prism software (one-way ANOVA or *t*-test; *p < 0.05; **p < 0.01; ***p < 0.001).

qRT-PCR analysis showed that both PRV WT and EP0 KO strains suppressed sp53 mRNA transcription to a similar extent, indicating that EP0 does not affect sp53 at the transcriptional level (Figure 6A). Treatment with the proteasome inhibitor MG132 partially inhibited sp53 F229V degradation during PRV infection (Figure 6B), suggesting that proteasome-mediated degradation contributes to this process. Ubiquitination assays further demonstrated that sp53 F229V ubiquitination levels were significantly higher in PRV WT-infected cells compared to EP0 KO-infected cells (Figure 6C), implicating EP0 in facilitating proteasome-dependent degradation of sp53 F229V.

These findings suggest that PRV employs a dual mechanism to reduce sp53 F229V protein levels: transcriptional suppression, might be the effect of the virion host shutoff protein (He et al., 2020) and EP0-mediated ubiquitination. The involvement of EP0 in proteasome-dependent degradation underscores its critical role in counteracting mutant sp53’s antiviral functions, thereby enhancing PRV replication.

## Discussion

This study revealed a complex interaction between porcine p53 and pseudorabies virus (PRV). We demonstrated that wild-type sp53 promoted PRV replication, and the sp53 F229V mutation significantly inhibited PRV replication, suggesting that this mutation confers antiviral properties. Based on data from this *in vitro* study, PRV reduced sp53 via two routes: transcriptional suppression and EP0-linked ubiquitination accompanied by proteasome-dependent turnover. These findings underscore the dual role of p53 in PRV infection: as a facilitator of replication in its wild-type form and a barrier in its mutant form.

The role of p53 in viral infections has been extensively studied, with most research highlighting its antiviral effects through the induction of apoptosis, regulation of DNA damage response or cell cycle arrest (Liu et al., 2013; Rivas et al., 2010). However, certain viruses have evolved mechanisms to exploit p53 for their replication. For instance, HSV-1 stabilizes wild-type p53 in the early stage of infection to promotes the expression of ICP27, thereby facilitating viral replication, yet p53’s negative actions are later antagonized by viral proteins (Aloni-Grinstein et al., 2018). There is also research indicating that p53 positively regulates PRV replication and pathogenesis both *in vitro* and *in vivo* (Li et al., 2019). Our research confirms that PRV stabilizes wild-type sp53 early during infection but utilizes EP0 to degrade sp53 F229V, effectively neutralizing its antiviral activity.

Our data, together with conflicting reports in the literature, prompt us to propose a time-resolved model of p53 regulation during PRV infection Early after entry (e.g., 0–6 hpi), stress and DDR-linked signaling can stabilize wild-type p53, coinciding with the onset of immediate-early/early viral programs (reported as “pro-viral” in some systems). Later (e.g., ≥ 6–12 hpi), accumulation of EP0 is consistent with ubiquitination-coupled proteasomal turnover of p53 species; this provides a route to counteract antiviral p53 variants such as sp53 F229V and maintain productive replication. Differences in sampling time, p53 genotype/status, cell context, MOI/synchrony, and readouts (total vs. PTM-specific p53) likely underlie the divergent conclusions across studies (Maruzuru et al., 2013).

EP0 contains an N-terminal RING-finger motif and is homologous to HSV-1 ICP0, a RING-type E3 ubiquitin ligase that promotes lytic replication by ubiquitinating numerous host substrates and antagonizing intrinsic/innate defenses. By analogy, one possibility is that EP0 functions as an intrinsic E3, whereas an alternative (not mutually exclusive) possibility is that EP0 recruits cellular E3s to promote sp53 ubiquitination. Consistent with this framework, ICP0 has been shown to catalyze ubiquitination in vitro with defined E2 partners and to target factors such as RNF8/RNF168 and p53, thereby remodeling antiviral and DNA-damage responses. While our data support proteasome-dependent turnover and increased ubiquitination of sp53 during PRV infection, determining whether EP0 acts as an E3 or engages host E3(s) will require structure–function analyses of EP0 (e.g., RING-disrupting mutants), lysine-mapping on sp53, and ubiquitination reconstitution with candidate E2/E3 components. These experiments are a priority for future work. Our findings highlight the critical role of EP0 in modulating host defenses during PRV infection. By promoting the ubiquitination and degradation of sp53 F229V through the proteasome pathway, EP0 facilitates PRV replication, likely aiding the virus in evading p53 F229V-mediated antiviral responses. The sp53 F229V mutation, on the other hand, represents a functional gain-of-characteristics, as it loses its transcriptional activity while acquiring antiviral properties. This functional adaptation may reflect an evolutionary response to viral pressure, enabling cells harboring mutant p53 to better resist viral replication.

Mutant porcine p53, unlike wild-type p53, does not favor the replication of herpesviruses HSV-1 and PRV; instead, it inhibits viral proliferation. Whether this negative regulatory effect on viruses is broad-spectrum remains unconfirmed. As the most frequently mutated gene in human tumors, p53 not only loses its tumor-suppressive function upon mutation but also gains various oncogenic capabilities. However, the impact of mutant p53 on viral infection remains largely unknown. Investigating how mutant p53 influences viral infections—particularly those involving oncolytic viruses used in cancer therapy—will be highly significant for understanding tumor development, progression, and improving antitumor treatments.

These insights provide a deeper understanding of how PRV manipulates p53 and contribute to the broader knowledge of host-pathogen interactions. They also raise intriguing questions about the evolutionary significance of p53 mutations and their potential role in shaping antiviral responses.

It is important to note that sp53 F229V was observed in long-term passaged porcine cell lines and, to our knowledge, has not been reported in natural porcine populations or tumors. Nonetheless, the phenotype illustrates that certain p53 alterations can uncouple transcriptional activity from antiviral effects, consistent with the idea that p53 mutations exert mutation-specific and context-dependent functions beyond tumor suppression. Given the diversity of p53 variants in human cancers, a systematic evaluation of common tumor-associated p53 mutants across distinct virus families will be necessary to determine whether antiviral effects are generalizable or restricted to particular mutations, cell types, and infection stages.

Despite the insights gained from this study, several limitations warrant consideration. First, all data in this study were generated *in vitro* using PK15, CRL-derived cells, and porcine liver cells; these models do not fully recapitulate the complexity of *in vivo* PRV infection. Accordingly, our conclusions are limited to in vitro contexts, and controlled infection studies in pigs—with longitudinal tissue sampling and ex vivo assays in primary porcine cells—will be required to establish the relevance of wild-type and F229V p53 to PRV pathogenesis. Second, although we demonstrate that EP0 promotes proteasome-dependent degradation and increased ubiquitination of sp53 F229V, the proximal ubiquitination machinery remains undefined. It will be important to determine whether EP0 functions as an intrinsic E3 ligase or instead recruits host E3(s), and to map the relevant lysine residues and adaptor factors. Third, the generalisability of the sp53 F229V phenotype across cell types, tissues, and virus families is unknown. Systematic testing of common tumor-associated p53 variants across alphaherpesvirus and non-alphaherpesvirus infections will be necessary to delineate mutation-specific and context-dependent effects. Finally, exploring the evolutionary and functional significance of p53 mutations in viral-host interactions could provide new insights into the co-evolution of host defenses and viral strategies.

By elucidating the dual role of p53 and the critical function of EP0 in PRV infection, this study advances our understanding of the intricate interplay between host antiviral defenses and viral countermeasures. These findings not only contribute to virology but also open avenues for the development of novel therapeutic strategies targeting p53 and its regulatory pathways in viral infections.

## Data Availability

The original contributions presented in the study are included in the article/Supplementary material, further inquiries can be directed to the corresponding authors.

## Footnotes

1
http://tools.genome-engineering.org
