# Supplementary material for: The role and mechanism of p53 F229V mutation in inhibiting pseudorabies virus replication
Source: Front Microbiol. 2025 Nov 26;16:1628916. doi: 10.3389/fmicb.2025.1628916 (PMC12689903; doi:10.3389/fmicb.2025.1628916)
Supplement: Supplementary file 1 [file Supplementary_file_1.docx]

Supplementary

Table-1 Sequence of primers used for luciferase assay and plasmids construction.

| Primer | Sequence(5’-3’) | Size（bp） |
| --- | --- | --- |
| NOXA-F | CAGGCTTGCCCCGGCAAGTTGACACTAGGCTTGCCCCGGCAAGTTGG |  |
| NOXA-R | AATTCCAACTTGCCGGGGCAAGCCTAGTGTCAACTTGCCGGGGCAAGCCTGGTAC |  |
| GPX1-F | CGGGCCAGACCAGACATGCCTACACTGGGCCAGACCAGACATGCCTG |  |
| GPX1-R | AATTCAGGCATGTCTGGTCTGGCCCAGTGTAGGCATGTCTGGTCTGGCCCGGTAC |  |
| PIDD-F | CAGGCCTGCCTGCGTGCTGGGACATGTCTACACTAGGCCTGCCTGCGTGCTGGGACATGTCTG |  |
| PIDD-R | AATTCAGACATGTCCCAGCACGCAGGCAGGCCTAGTGTAGACATGTCCCAGCACGCAGGCAGGCCTGGTAC |  |
| GADD45-F | CGAACATGTCTAAGCATGCTGACACTGAACATGTCTAAGCATGCTGG |  |
| GADD45-F | AATTCCAGCATGCTTAGACATGTTCAGTGTCAGCATGCTTAGACATGTTCGGTAC |  |
| BAX-F | CTCACAAGTTAGAGACAAGCCTACACTTCACAAGTTAGAGACAAGCCTG |  |
| BAX-R | AATTCAGGCTTGTCTCTAACTTGTGAAGTGTAGGCTTGTCTCTAACTTGTGAGGTAC |  |
| MMP2-F | CAGACAAGCCTGAACTTGTCTACACTAGACAAGCCTGAACTTGTCTG |  |
| MMP2-R | AATTCAGACAAGTTCAGGCTTGTCTAGTGTAGACAAGTTCAGGCTTGTCTGGTAC |  |
| KILLER-F | CGGGCATGTCCGGGCAAGACGACACTGGGCATGTCCGGGCAAGACGG |  |
| KILLER-R | AATTCCGTCTTGCCCGGACATGCCCAGTGTCGTCTTGCCCGGACATGCCCGGTAC |  |
| PAI-F | CACACATGCCTCAGCAAGTCCACACTACACATGCCTCAGCAAGTCCG |  |
| PAI-R | AATTCGGACTTGCTGAGGCATGTGTAGTGTGGACTTGCTGAGGCATGTGTGGTAC |  |
| CDKN1A-F | CGAACATGTCCCAACATGTTGACACTGAACATGTCCCAACATGTTGG |  |
| CDKN1A-R | AATTCCAACATGTTGGGACATGTTCAGTGTCAACATGTTGGGACATGTTCGGTAC |  |
| PUMA-F | CCTGCAAGTCCTGACTTGTCCACACTCTGCAAGTCCTGACTTGTCCG |  |
| PUMA-R | AATTCGGACAAGTCAGGACTTGCAGAGTGTGGACAAGTCAGGACTTGCAGGGTAC |  |
| MDM2-F | CGGTCAAGTTGGGACACGTCCACACTGGTCAAGTTGGGACACGTCCG |  |
| MDM2-R | AATTCGGACGTGTCCCAACTTGACCAGTGTGGACGTGTCCCAACTTGACCGGTAC |  |
| sp53-F | GACGATGACAAGGGATCCATGGAGGAGTCGCAGTCC | 1182 |
| sp53-R | GCCATGGCGGCCAAGCTTTCAGTCTGAGTCAGGTCC |  |
| pRK5-UL12-F | GACGATGACAAGGGATCCATGGCGGCGCTCGTTTTG | 1452 |
| pRK5-UL12-R | GCCATGGCGGCCAAGCTTTTAGATCACGACGCGGTTGCTGG |  |

Table-2 Sequence of primers used for generation of PK15 (p53^-/-^) cells

| Primer | Sequence (5’-3’) | Size（bp） |
| --- | --- | --- |
| sp53-target 1-F | CACCGAGGAGTCGCAGTCCGAGCT |  |
| sp53-target 1-R | AAACAGCTCGGACTGCGACTCCTC |  |
| sp53-target 2-F | CACCGTCATCCAGCCAGTTCGTGAC |  |
| sp53-target 2-R | AAACGTCACGAACTGGCTGGATGAC |  |
| sp53-target 3-F | CACCGGCACTCTGGAGGCGTCATC |  |
| sp53-target 3-R | AAACGATGACGCCTCCAGAGTGCC |  |
| sp53-target 1-F (identification) | GGGTGGACAATCCCTCTCTG | 478 |
| sp53-target 1-R (identification) | CAGTCCTTACCAGCAGGTTGT |  |
| sp53-target 2/3-F (identification) | TGTGAGTGGAGTCTCAAGGGA | 610 |
| sp53-target 2/3-R (identification) | CTGACACAAAGCCAAAGGCTG |  |

Table 3. Primers used for Real-time PCR.

| Primer | Sequence(5’-3’) | Size（bp） |
| --- | --- | --- |
| sp53-F(RT) | ACCACCATCCACTACAACTTCA | 137 |
| sp53-R(RT) | CAGGACAGGCACAAACACG |  |
| sp21-F(RT) | GCAGACCAGCATGACAGATTT | 168 |
| sp21-R(RT) | AAGGCTTCCAGCAGCCTAGT |  |
| sGAPDH-F(RT) | TGAAGGTCGGAGTCAACGGATTTGGT | 173 |
| sGAPDH-R(RT) | CATGTGGGCCATGAGGTCCACCAC |  |

**Supplementary Fig. S1**


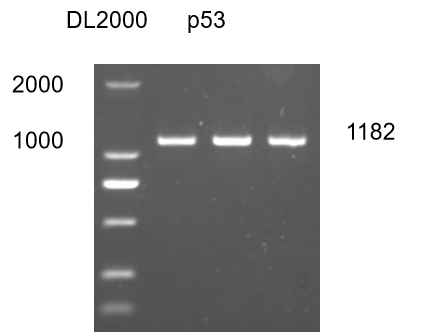

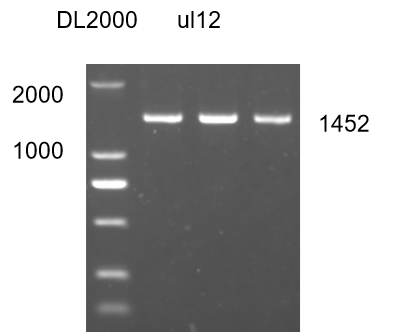


**Fig. S1** PCR amplification of p53 and UL12 fragments. Lanes show expected bands at 1182 bp for p53 and 1452 bp for UL12.

**Supplementary Fig. S2**


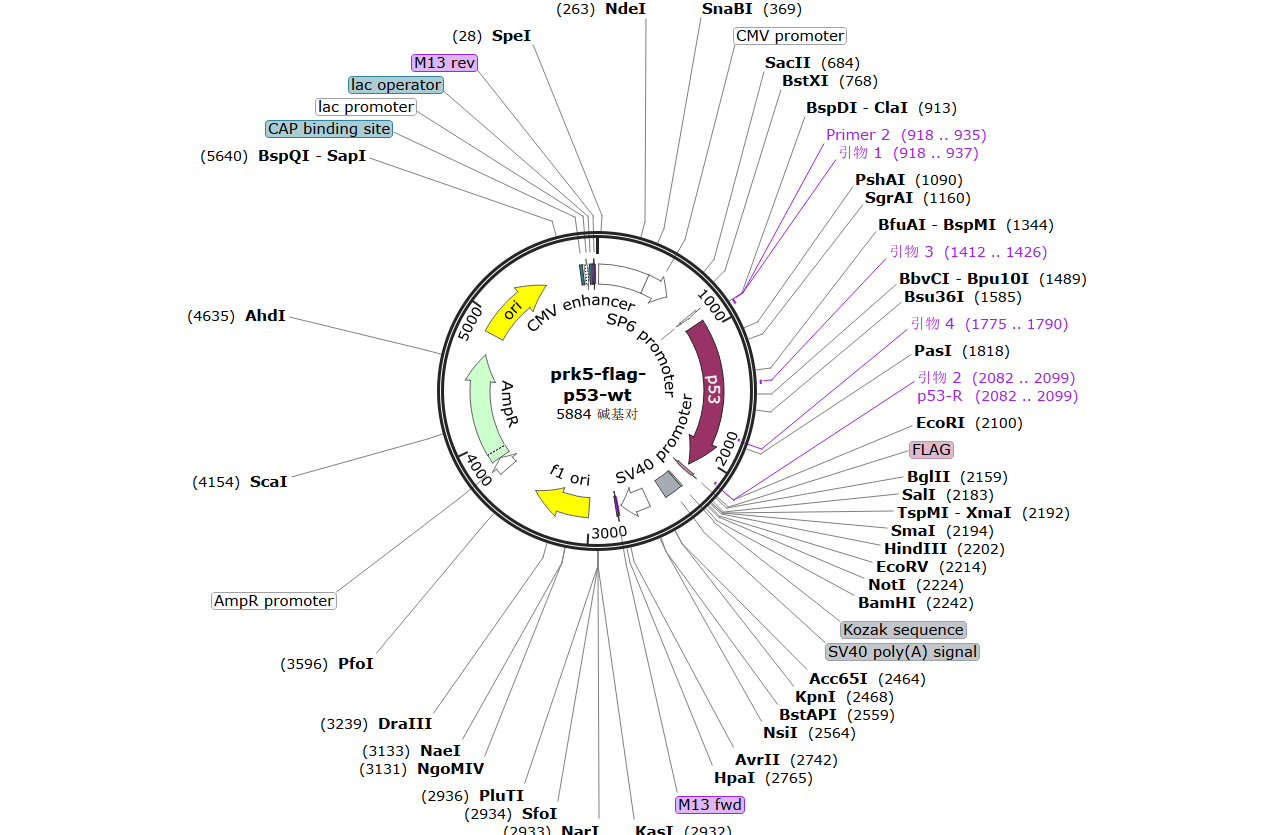


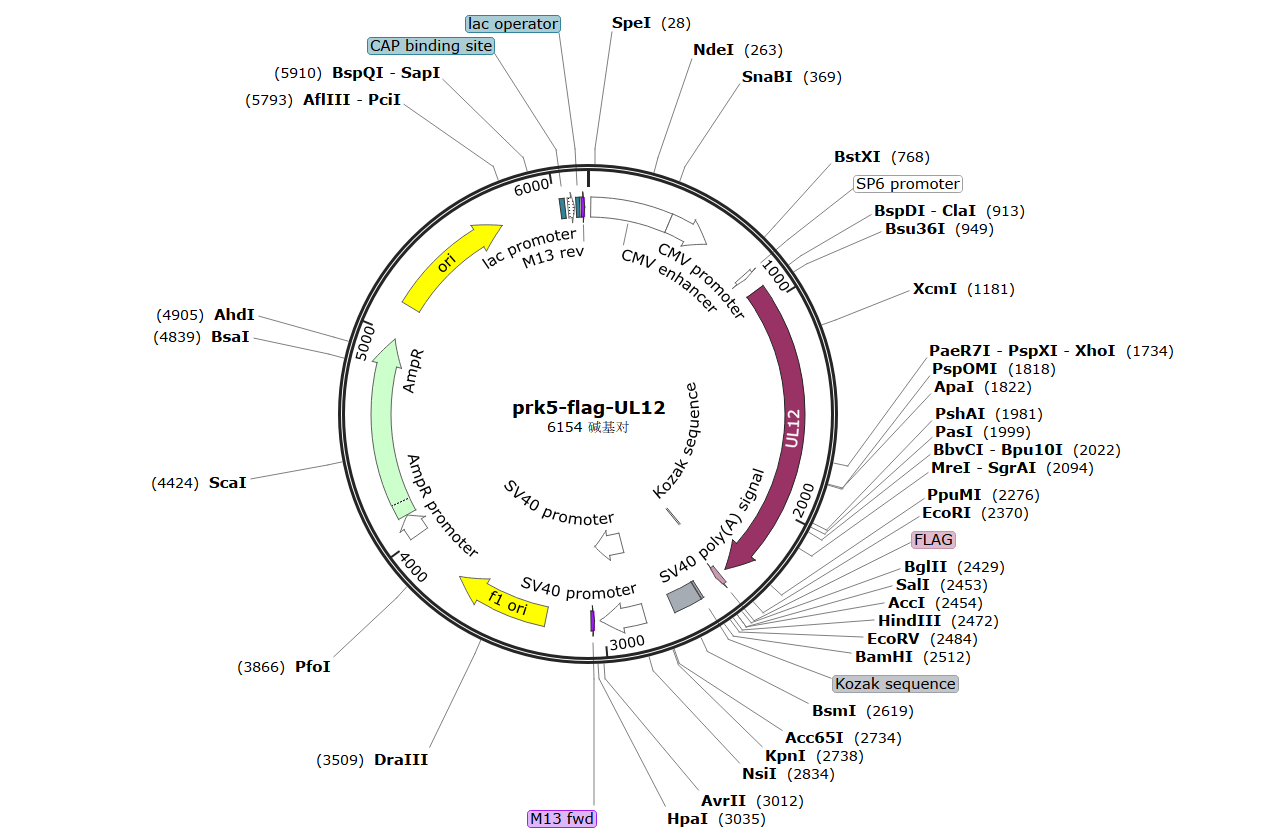


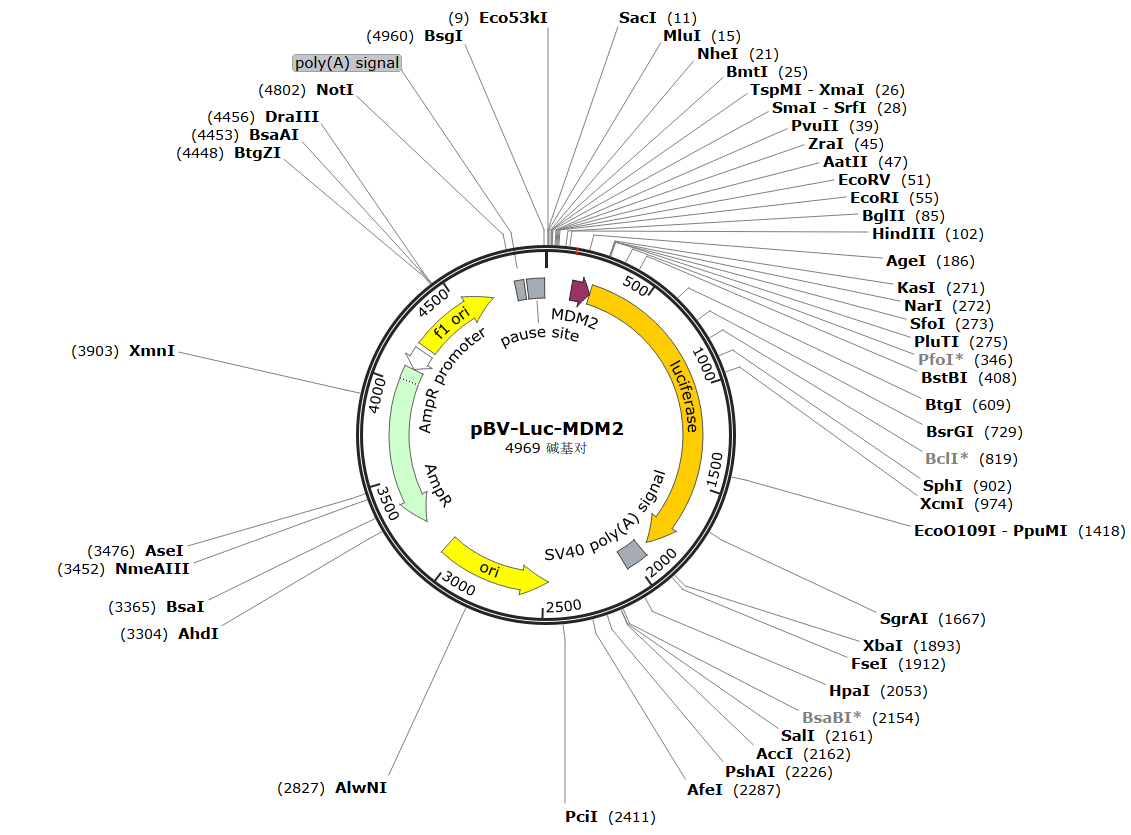


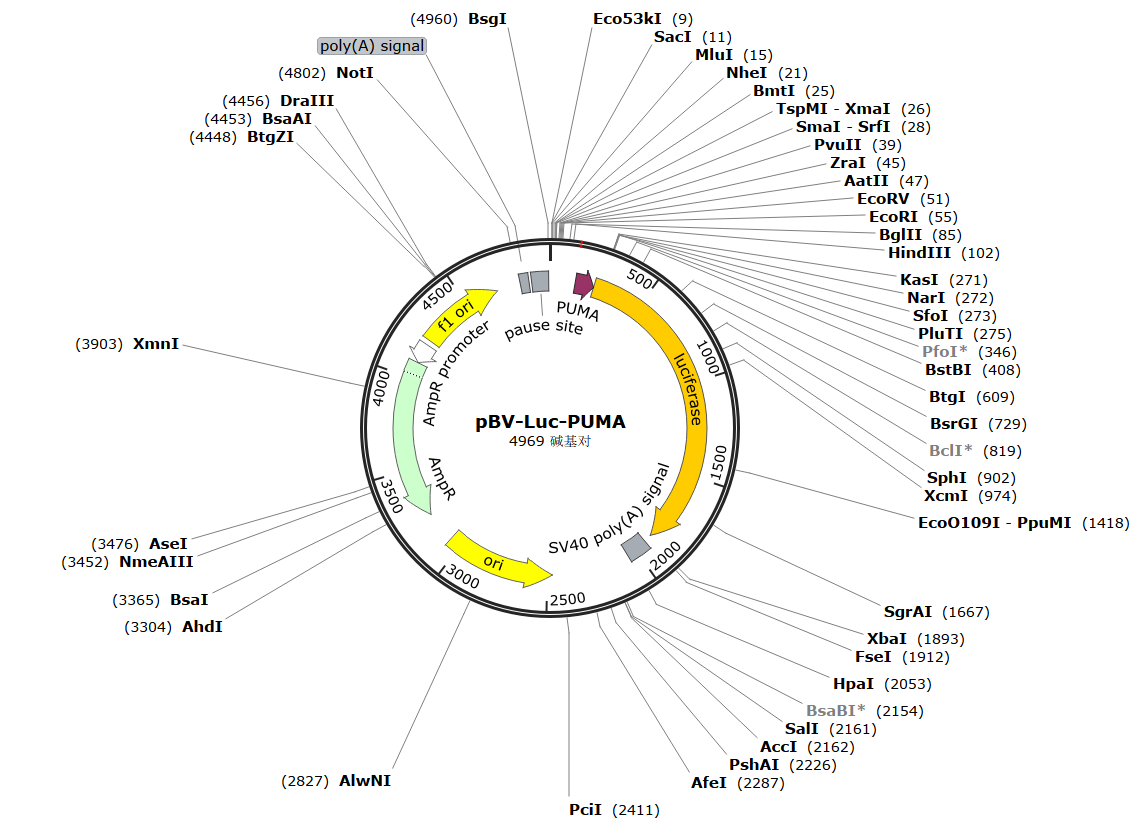


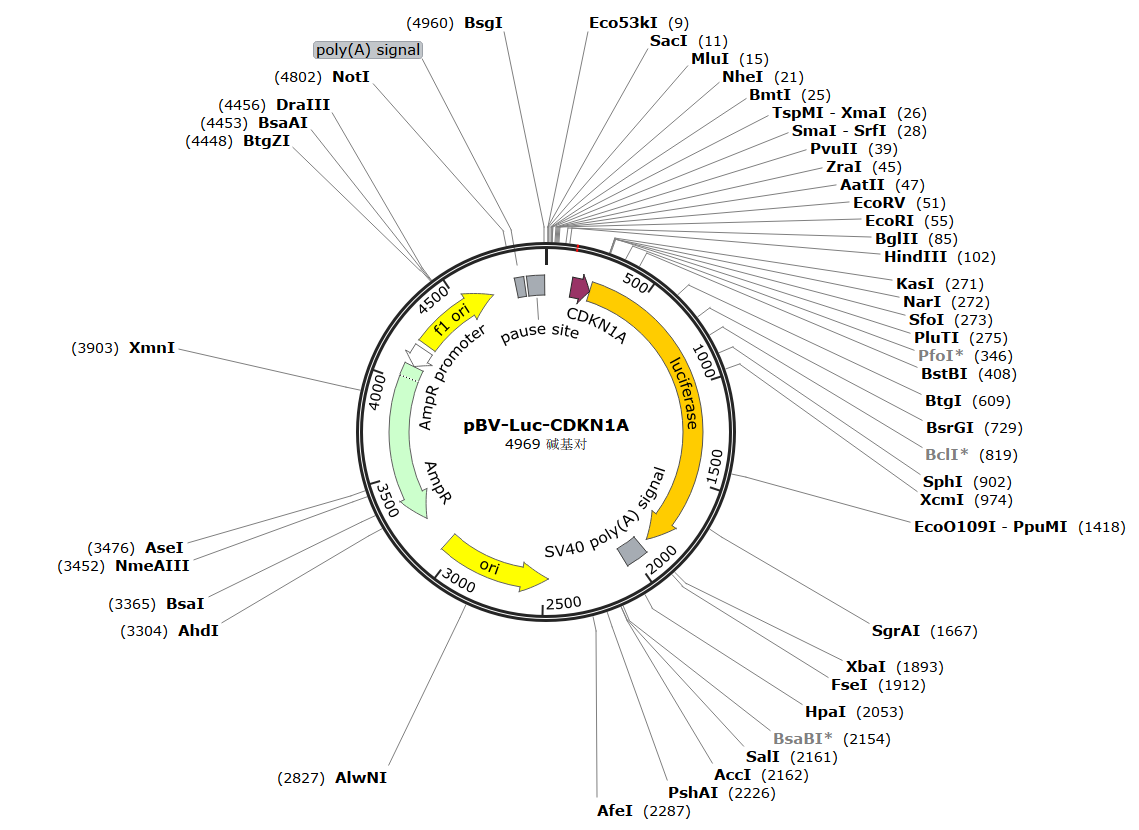


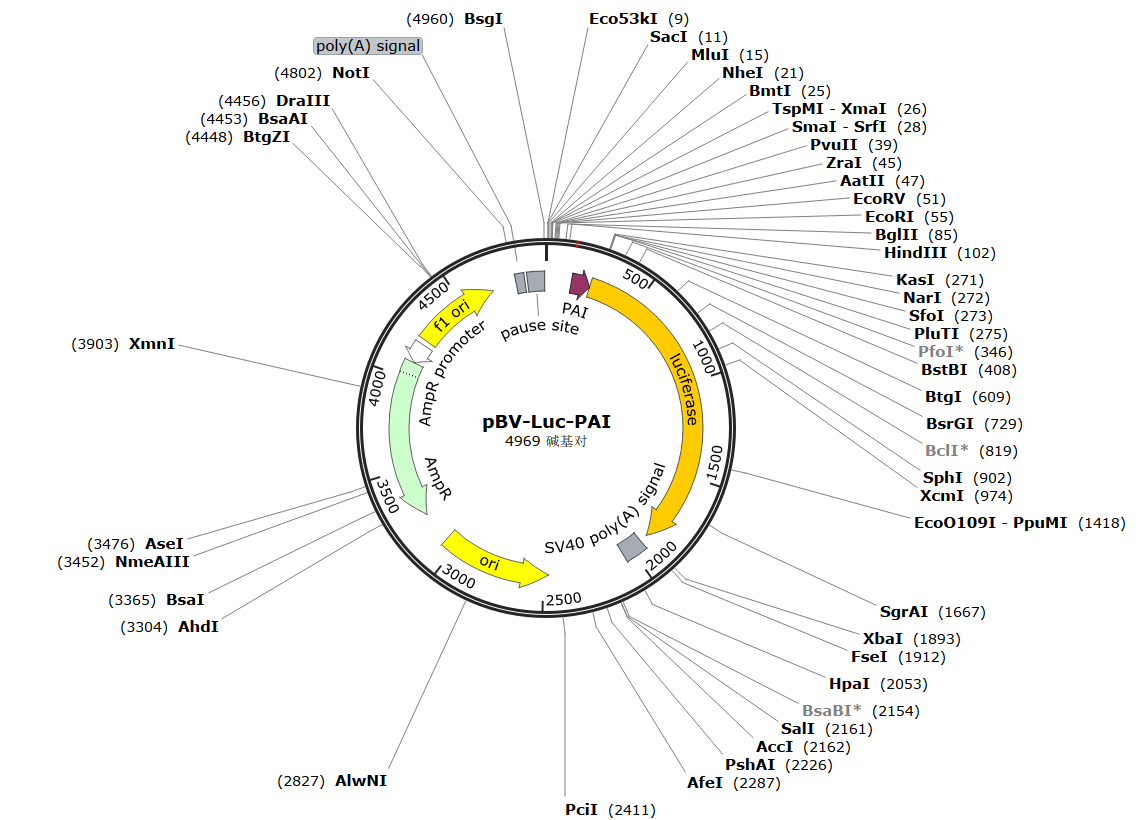


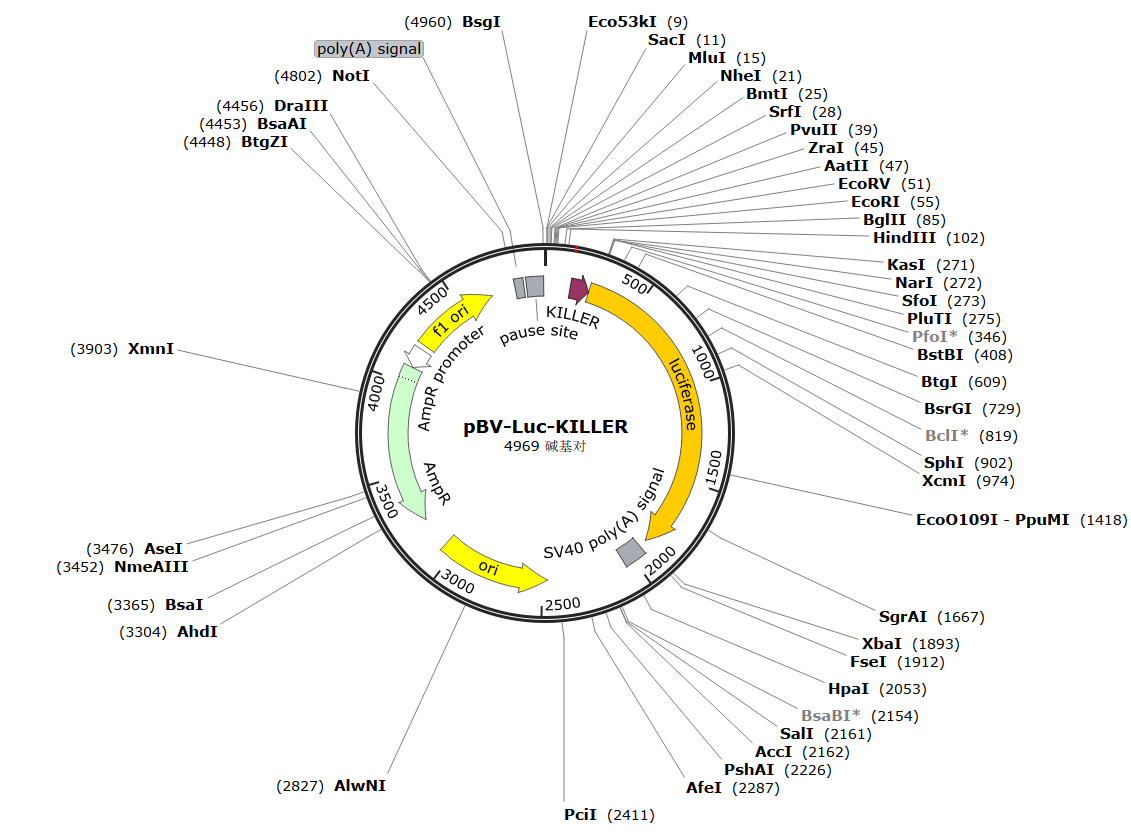


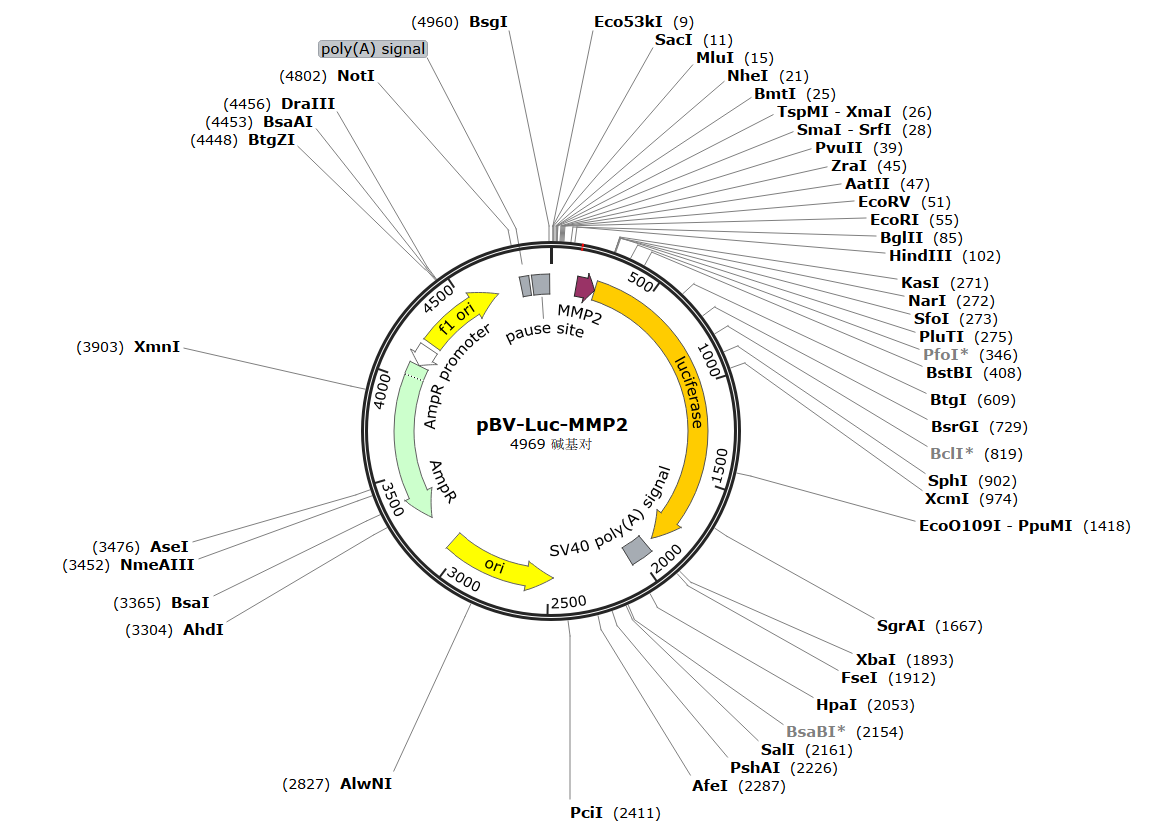


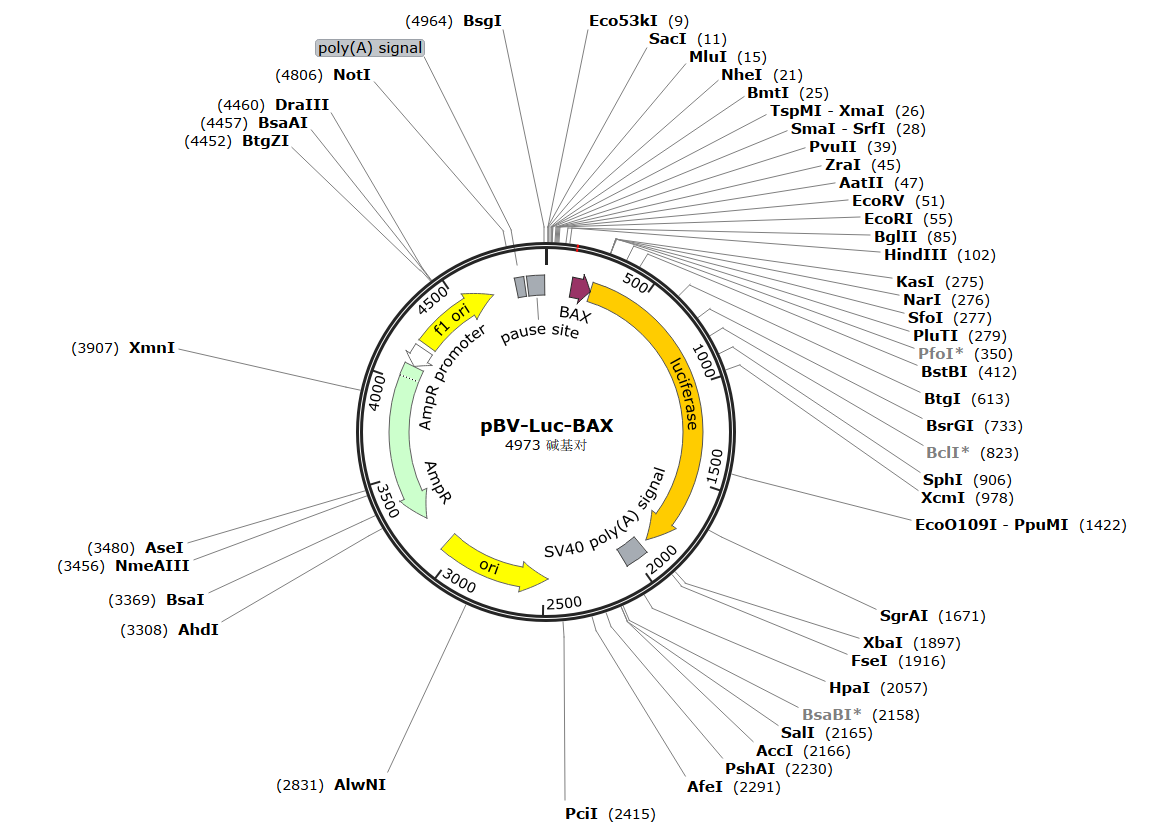


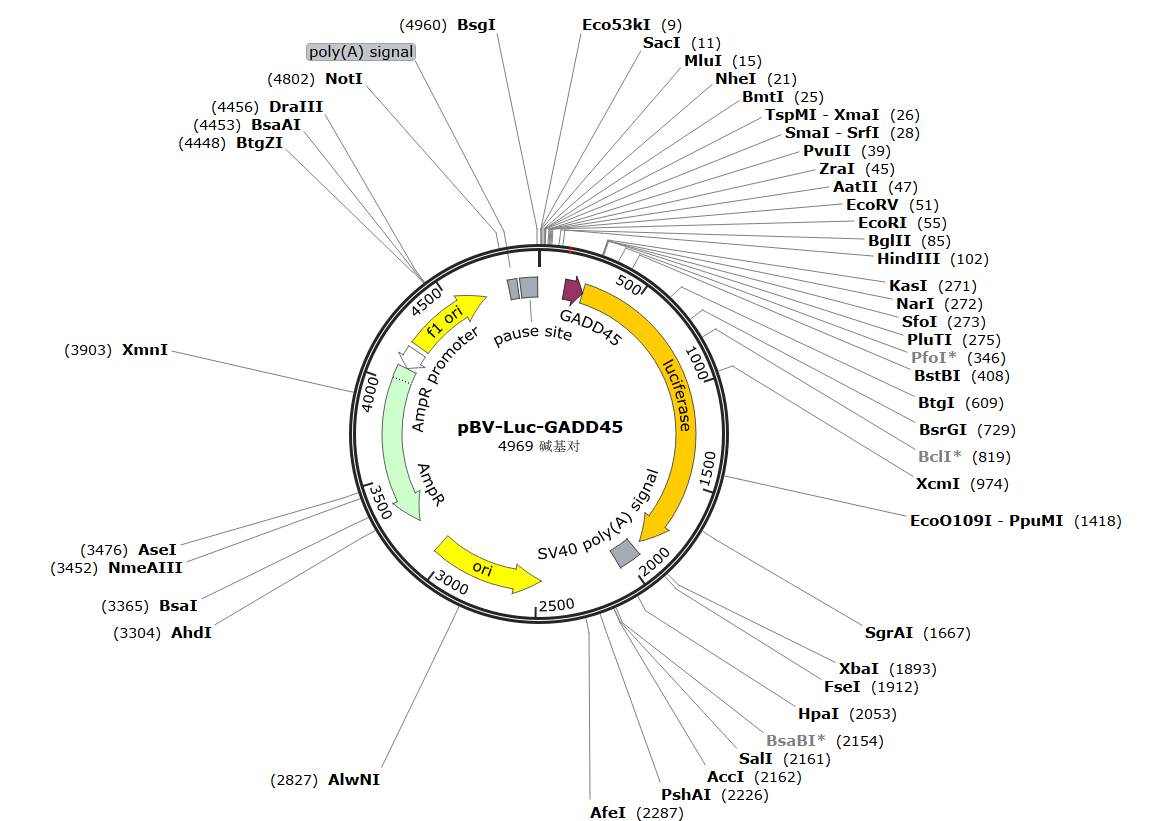


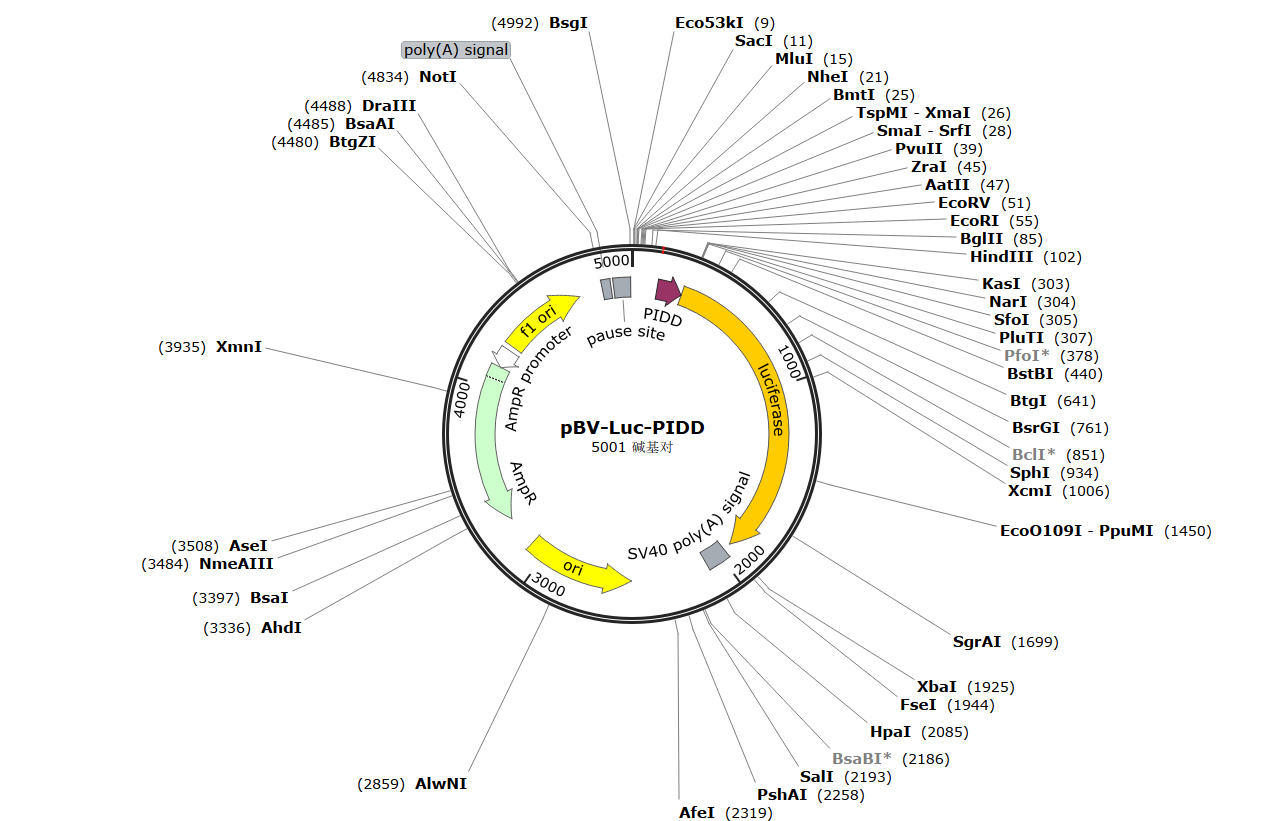


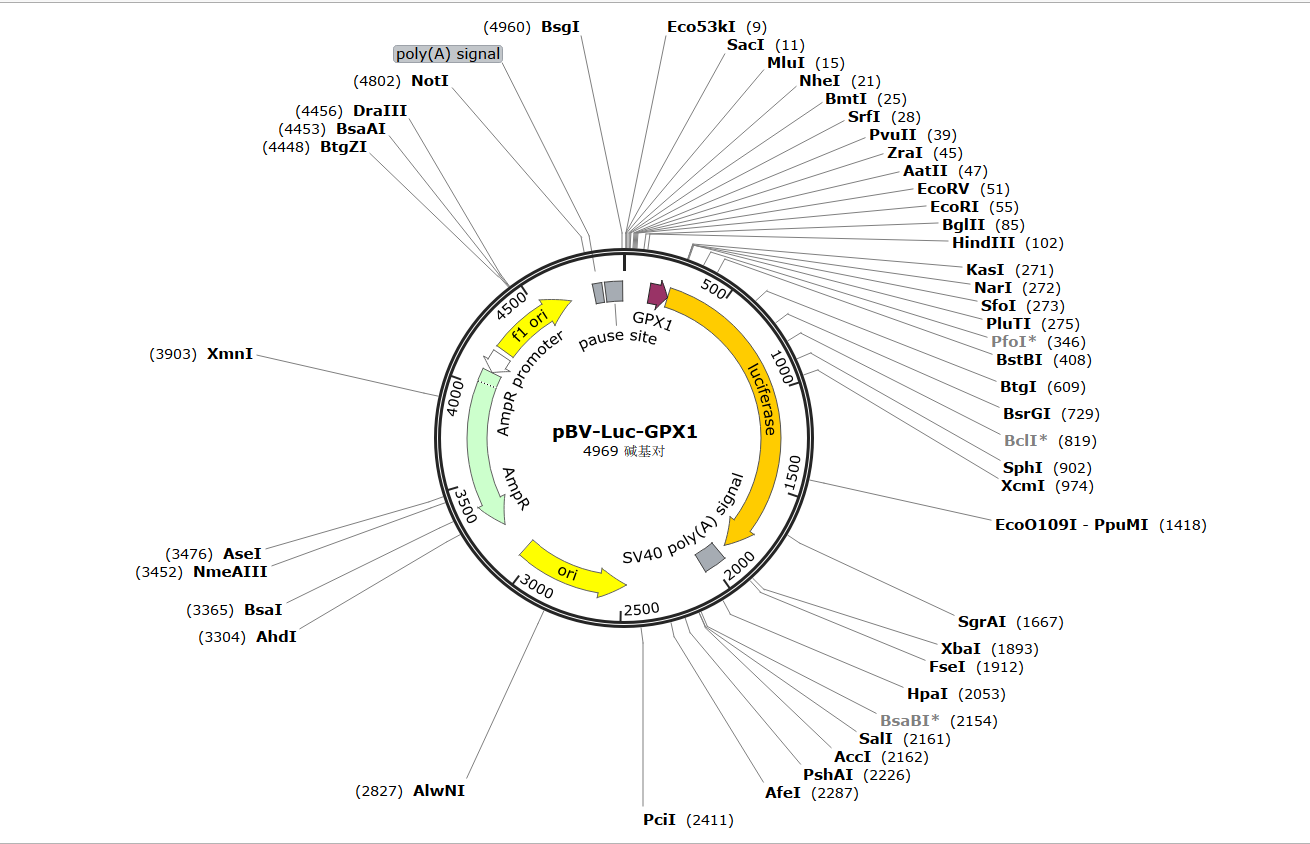


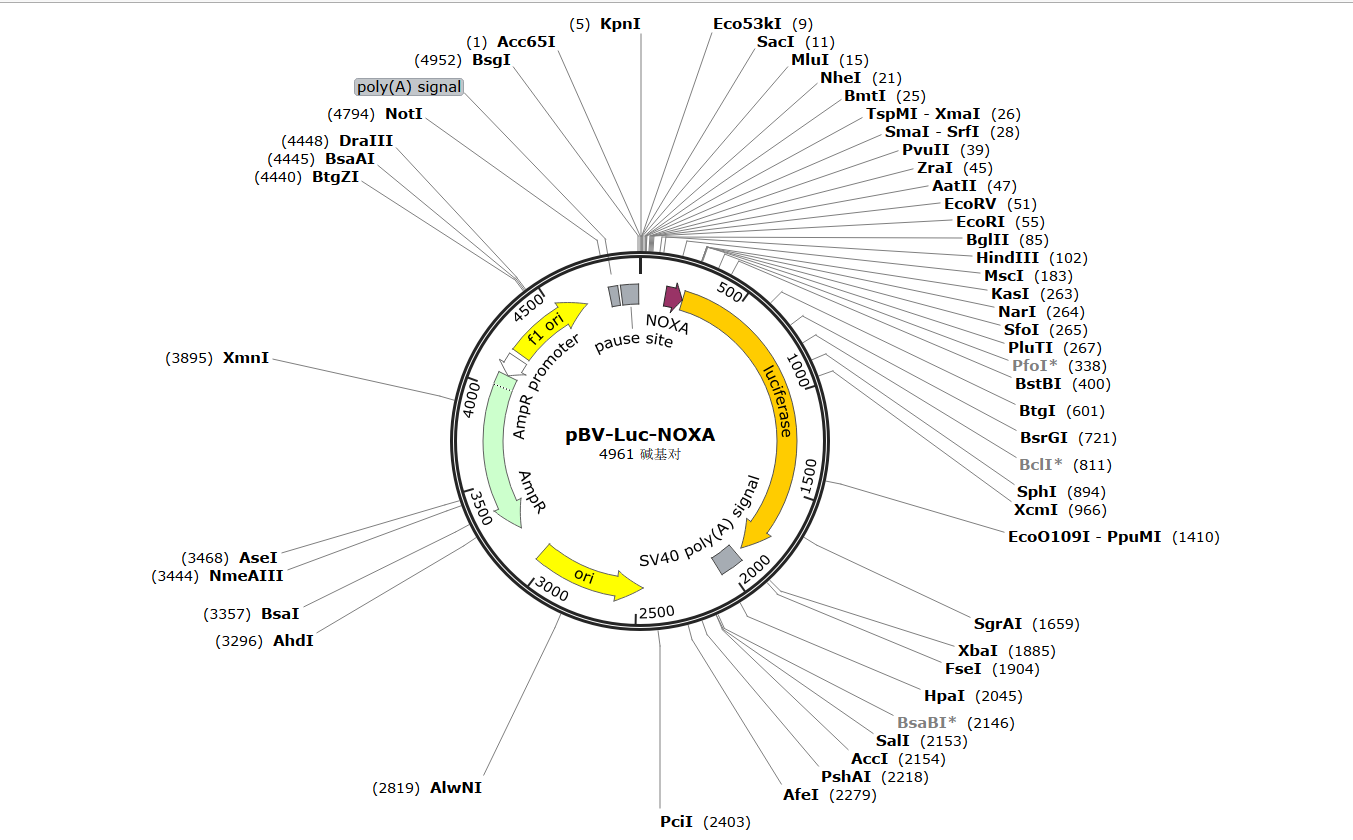


**Fig. S2** Map of plasmid constructs.

**Supplementary Fig. S3**


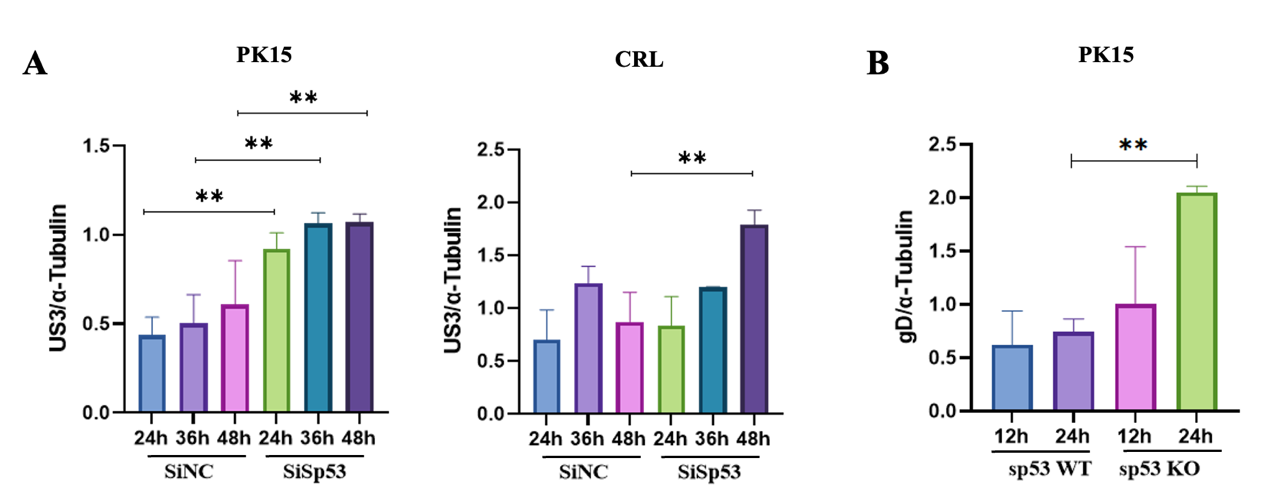


**Fig. S3** Relative Expression Levels of Viral Protein in PK15 and CRL cells after knocking down or knocking out sp53 F229V. (A) PK15 and CRL cells were transfected with either a non-targeting control siRNA (Si-NC) or an siRNA targeting sp53 (Si-sp53) for 24 hours, followed by infection with PRV WT (MOI = 0.1). Cells were collected at 24-, 36-, and 48- hours post-infection for Western blot analysis of viral protein expression. Band intensities were quantified using ImageJ software and normalized to α-Tubulin. (B) PK15 cells with or without sp53 were infected by PRV WT (MOI=1) for 12 and 24 h, the cell lysate was collected for Western blot analysis of viral protein expression. Band intensities were quantified using ImageJ software and normalized to α-Tubulin. Data represent mean ± SD of three independent experiments. **P* < 0.05, ***P* < 0.01, ****P* < 0.001.

**Supplementary Fig. S4**


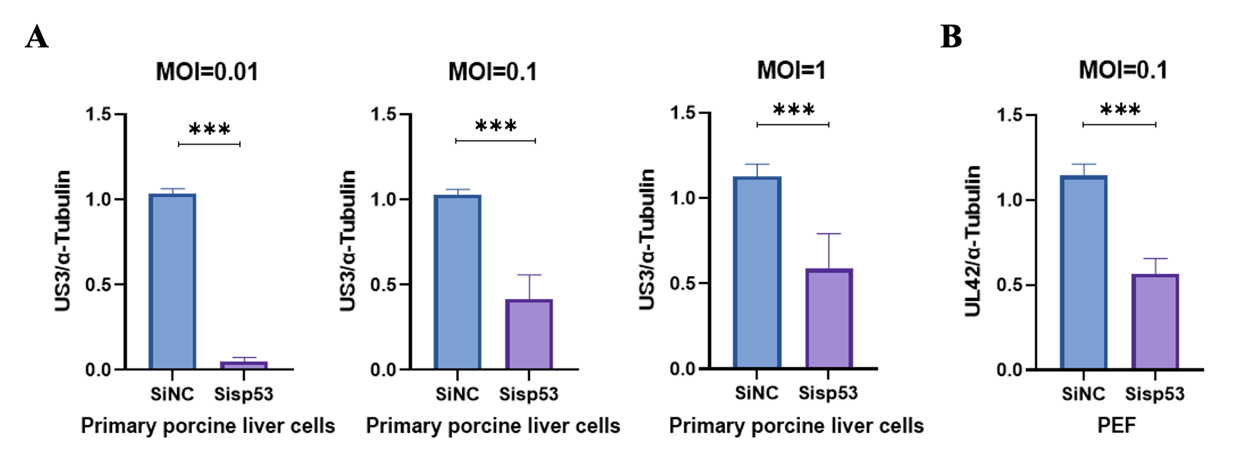


**Fig. S4** Relative expression levels of viral protein in Primary porcine liver cells and PEF cells after knocking down wild-type sp53. (A) Primary porcine liver cells transfected with SiNC or Si-sp53 for 24 hours, and then infected with PRV WT (MOI=0.01, 0.1 or 1) for 24 h, Western blot was performed to analyze the expression of viral proteins. Band intensities were quantified using ImageJ software and normalized to α-Tubulin. (B) PEF cells transfected with SiNC or Si-sp53 for 24 hours, and then infected with PRV WT (MOI= 0.1) for 24 h, Western blot was performed to analyze the expression of viral proteins. Band intensities were quantified using ImageJ software and normalized to α-Tubulin. Data are shown as mean ± SD of three independent experiments. Statistical analysis was performed by Student's t-test. ****P* < 0.001.

**Supplementary Fig. S5**


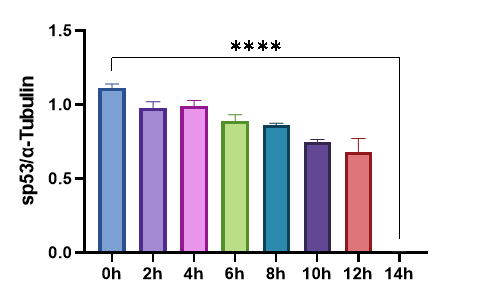


**Fig. S5** Relative Expression Levels of sp53 F229V in PK15 cells after PRV infection. PK15 cells were infected with PRV WT (MOI=1), and cells were harvested at 2-, 6-, 8-, 10-, 12-, and 14- hours post-infection for Western Blot analysis of sp53 protein expression. Band intensities were quantified using ImageJ software and normalized to α-Tubulin. Data are shown as mean ± SD of three independent experiments. Statistical analysis was performed by Student's t-test. ****P* < 0.001.
